# Optical meta-waveguides for integrated photonics and beyond

**DOI:** 10.1038/s41377-021-00655-x

**Published:** 2021-11-22

**Authors:** Yuan Meng, Yizhen Chen, Longhui Lu, Yimin Ding, Andrea Cusano, Jonathan A. Fan, Qiaomu Hu, Kaiyuan Wang, Zhenwei Xie, Zhoutian Liu, Yuanmu Yang, Qiang Liu, Mali Gong, Qirong Xiao, Shulin Sun, Minming Zhang, Xiaocong Yuan, Xingjie Ni

**Affiliations:** 1 State Key Laboratory of Precision Measurement Technology and Instruments, Department of Precision Instrument, Tsinghua University, 100084 Beijing, China; 2 Shanghai Engineering Research Center of Ultra-Precision Optical Manufacturing and School of Information, Science and Technology, Fudan University, Shanghai, 200433 China; 3 School of Optical and Electronic Information, Huazhong University of Science and Technology, Wuhan, 430074 China; 4 Department of Electrical Engineering, Pennsylvania State University, University Park, PA 16802 USA; 5 Optoelectronic Division, Department of Engineering, University of Sannio, I-82100 Benevento, Italy; 6 Department of Electrical Engineering, Stanford University, Stanford, CA 94305 USA; 7 Nanophotonics Research Centre, Shenzhen Key Laboratory of Micro-Scale Optical Information Technology, Shenzhen University, Shenzhen, 518060 China; 8 Key Laboratory of Photonic Control Technology, Ministry of Education, Tsinghua University, 100084 Beijing, China; 9 Yiwu Research Institute of Fudan University, Chengbei Road, Yiwu City, 322000 Zhejiang China; 10 Wuhan National Laboratory for Optoelectronics, Huazhong University of Science and Technology, Wuhan, 430074 Hubei China

**Keywords:** Metamaterials, Silicon photonics, Nanophotonics and plasmonics, Transformation optics

## Abstract

The growing maturity of nanofabrication has ushered massive sophisticated optical structures available on a photonic chip. The integration of subwavelength-structured metasurfaces and metamaterials on the canonical building block of optical waveguides is gradually reshaping the landscape of photonic integrated circuits, giving rise to numerous meta-waveguides with unprecedented strength in controlling guided electromagnetic waves. Here, we review recent advances in meta-structured waveguides that synergize various functional subwavelength photonic architectures with diverse waveguide platforms, such as dielectric or plasmonic waveguides and optical fibers. Foundational results and representative applications are comprehensively summarized. Brief physical models with explicit design tutorials, either physical intuition-based design methods or computer algorithms-based inverse designs, are cataloged as well. We highlight how meta-optics can infuse new degrees of freedom to waveguide-based devices and systems, by enhancing light-matter interaction strength to drastically boost device performance, or offering a versatile designer media for manipulating light in nanoscale to enable novel functionalities. We further discuss current challenges and outline emerging opportunities of this vibrant field for various applications in photonic integrated circuits, biomedical sensing, artificial intelligence and beyond.

## Abstract (web summary)

Recent years have witnessed substantial potential in allying meta-optics with diverse waveguide platforms to enable exotic manipulation of guided light signals. This review cataloged recent advances on meta-waveguides for photonic integration.

## Graphical abstract

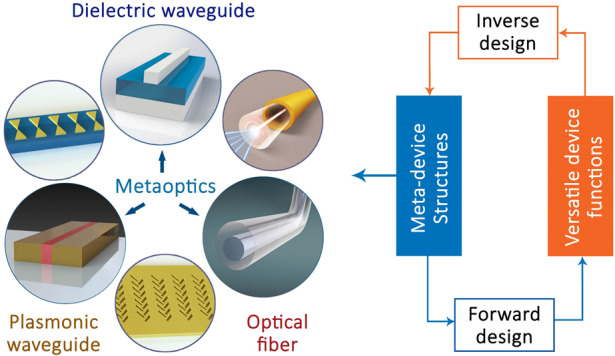

## Introduction

Efficient manipulation of guided electromagnetic waves is of vital significance in numerous applications in nanophotonics and integrated optics. Distinctive from its electronic counterparts, photonic integrated circuits deploy guided light waves instead of electrical signals to carry information^1,2^. As the bandwidth and power density limit of data transport in electrical wires are increasingly manifesting with higher integration density^3^, integrated optical scenarios have shown promising inroads towards ultrafast and broadband information processing with low power consumption that may potentially circumvent current electrical bottleneck^4–6^. Various applications have been reported in this field such as chip-scale optical signal processing, communications, and analog computing^7–9^, as well as emerging technologies in quantum, biomedicine, and sensing^10,11^.

However, recent advancements of photonic integrated circuits are hindered by limitations in its most fundamental building block of traditional optical waveguides, in terms of restrained accessible functionalities, compromised efficiency and bulk footprint^12–14^. The very limited design library of conventional waveguide structures substantially constraints their function- alities^12,15^ to mostly mere waveguiding. For instance, dielectric waveguides are ubiquitously applied to control on-chip propagating modes, while plasmonic waveguides play an essential role in guiding surface waves. Optical fibers are ideal for long-distance information communications and optical connections between chips and boards. However, the continuous trend towards miniaturized and versatile photonic systems demands more complicated device functions to be realized in a compact, multifunctional, configurable and CMOS-compatible way^1^.

Fulfillment of these tasks will entail novel waveguide structures. Fortunately, recent advent of metasurfaces and metamaterials opens a new pathway towards powerful light manipulation by engineering photonic structures in subwavelength scale^14,15^. Transferring the concept of meta-optics into guided waves can help overcome the abovementioned challenges^12^, by infusing new degrees of freedom into waveguide landscapes to dramatically boost device performance and enable novel functionalities^12–16^.

Optical metasurfaces and metamaterials are generally composed of judiciously designed artificial structures with feature size much smaller than light wavelength^16,17^. Metamaterials constructed by three-dimensional bulk subwavelength architectures can realize spatially changing exotic optical parameters (including the permittivity and permeability), giving rise to the transformation optics for invisibility cloaks and slow light phenomena^18–20^. Metasurfaces, in contrast, applying two-dimensional arrays of scatterers^21–23^, also exhibit unprecedented flexibility in controlling the fundamental attributes of electromagnetic waves, such as the amplitude, phase, polarization, wavefront, and so on. Fruitful applications are also reported such as metalens^24–26^, efficient holograms^27,28^, functional coatings^29^, color display^30,31^, LiDAR^32^ and nonlinear optics^33,34^. Previous research attentions are mainly devoted to free-space applications. However, recent years have seen a tremendous interest in synergizing meta-optics with various optical waveguides to largely empower conventional photonic devices^12–14,35–110^. The advancement of subwavelength meta-structured waveguides can not only extend meta-optics physics to the realm of guided electromagnetic waves, but also promise to reshape the landscapes of photonic integrated circuits and massive emergent applications such as lab-on-chip technologies and neuromorphic photonics^8,12,13,59,60^.

In this review, we discuss recent progress on various subwavelength meta-structured waveguides, encompassing a broad class of photonic devices and systems that ally metamaterials and metasurfaces with diverse optical waveguides (dielectric/plasmonic/optical fibers)^35–110^. Brief physical fundamentals with explicit design methods and representative applications for meta-waveguides are comprehensively summarized. We highlight how incorporating the concepts of meta-optics with waveguide technologies can propel photonic integrated circuits into new heights, by providing versatile efficient coupling interfaces^35–50^, novel on-chip optical signal processing paradigms^12,51–73^ and diverse platforms for sensing, imaging and artificial intelligence^57–60,74–76^. We further comment on current challenges in device design and practical hurdles from ripening into viable technology. Potential future research directions are also discussed based on current perspectives.

## Meta-waveguide fundamentals and properties

**Fig. 1:**
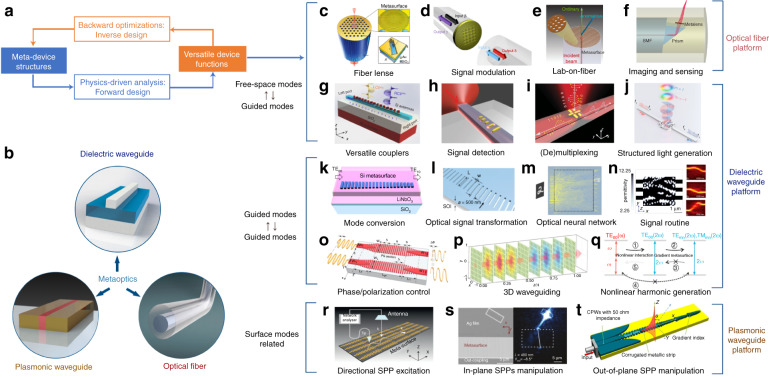
Allying metasurfaces and metamaterials with different waveguide platforms for various photonic applications. **a** If classified by design method, meta-waveguides can be conceived either by physics intuition-based forward design or computer optimizations-based inverse design. **b** If categorized by waveguide platforms, meta-optics can be saddled with dielectric waveguides, optical fibers and plasmonic waveguides. **c**–**t** Brief zoology of meta-waveguides. A plethora of applications are prototyped by the playground of meta-waveguides, including, for instance, metasurface-patterned dielectric waveguides^35–44,50–53,82^, forward-designed metamaterial waveguides^54–56,83–89^, metasurface- or metamaterial-hybrid optical fibers^74–76,96–110^, meta-structures- assisted plasmonic waveguides^70–73,112–128^, inverse-designed metamaterial waveguides^47,57–69^ and etc.^535,536^. Panels adapted from: De Gruyter (**c**)^100^; Springer Nature (**d**^96^, **e**^75^, **f**^74^, **j**^47^, **k**^51^, **l**^53^, **p**^535^, **q**^52^, **r**^70^, **s**^124^); OSA (**g**^44^, **m**^59^, **n**^58^, **o**^56^); ACS (**h**)^37^; AAAS (**i**)^50^; AIP Publishing (**t**)^127^.

As is illustrated in Fig. 1a, b, meta-waveguides can be classified via either design methods or underpinning waveguide platforms^12,13^:

- On the one hand, meta-waveguides can be conceived by physical intuition-based approaches (namely forward design henceforth) by leveraging the toolbox of metasurfaces and metamaterials with waveguide optics (discussed in Sections “Dielectric waveguide-integrated meta-structures”, “Optical meta-fibers”, and “Plasmonic meta-devices for controlling surface waves”).
- On the other hand, we can also use inverse design^15^, which relies on computer optimizations, to develop free-formed analog (curvilinear boundaries)^57–60^ or digital metamaterial waveguides^61–69,111^ (elaborated in Section “Inverse-designed metamaterial waveguides”).

Based on different waveguide platforms, metasurfaces and metamaterials can be synergized with dielectric waveguides and optical fibers to tailor optical modes, or with plasmonic waveguides to manipulate surface waves, as shown in Fig. 1b.

- By saddling subwavelength architectures on top of dielectric waveguides, exquisite control over the electromagnetic fields is attainable to facilitate versatile couplers^36–44^, compact polarization- or wavelength- routers^48–50^, on-chip structured light generators^35,45–47,77–81^, integrated mode convertors^51,82–89^, sensors^90–93^ and nonlinear devices^52,94,95^ (discussed in Section “Dielectric waveguide-integrated meta-structures”).
- The introduction of meta-structures into optical fibers also greatly enriches traditional fiber devices, ushering the creation of tremendous novel meta-fiber applications in information modulations^96–98^, beam transformations^99–103^, imaging^74^ and numerous high-performance biochemical sensors and detectors^75,76,104–110^ (detailed in Section “Optical meta-fibers”).
- Meanwhile, meta-optics are hatching as excellent candidates to construct plasmonic meta-waveguides for controlling surface waves. Abundant research has been invigorated, including efficient multifunctional surface waves excita- tions^70,112–122^ and manipulations^71–73,123–128^ with largely miniaturized device footprint (detailed in Section “Plasmonic meta-devices for controlling surface waves”).

In the following, we start by briefly introducing the fundamentals and unique features of subwavelength meta-structured waveguides. General design process and device operation scenarios are concisely covered. Next, we comprehensively review latest researches derived from three different types of forward-designed meta-waveguides in separate sections, according to three distinctive underpinning waveguide platforms: dielectric waveguides, optical fibers and plasmonic waveguides (shown as Fig. 1b). Then we catalog inverse-designed metamaterial waveguides, focusing on waveguide-based design tutorials and algorithms, key applications, and comparisons of different design approaches for tailoring guided wave. Finally, we discuss current challenges and outline exciting opportunities of this vibrant field for integrated photonics and beyond.

### Definitions, features and properties

Meta-waveguides here refer to a set of physical structures with engineered subwavelength features that guide electromagnetic waves. A simple case is the waveguiding media itself is man-made metamaterials^129–133^ consisting of intricate subwavelength building blocks. Leveraging the mathematical technique called transformation optics^20^, researchers can design waveguides with artificial refractive index distribution to realize exotic waveguiding phenomena such as theoretical arbitrary waveguide bend and light trapping^129–135^. However, in addition to the loss issues, these scenarios encounter experimental challenges in fabricating sophisticated bulk structures at high optical frequencies^17,20^, prohibiting its widespread implementation.

Another general case is applying meta-structures on waveguide surfaces to act as Mie resonators or Rayleigh scatterers^21–23^ to perform designer index perturbations^35–52,136^. The structures can be metal or dielectric materials on top of as-fabricated waveguides after deposition and lift-off^35,37,48–52^. Alternatively, these subwavelength features (either fully^38,39,47,54–56,63–68^ or partially etched^45,78,84–89^) itself can be part of the waveguides defined by lithography. These devices are application-wise more appealing, for they are promising for mass-production by lithographic or imprinting-based nanopatterning^12,137–139^.

**Fig. 2:**
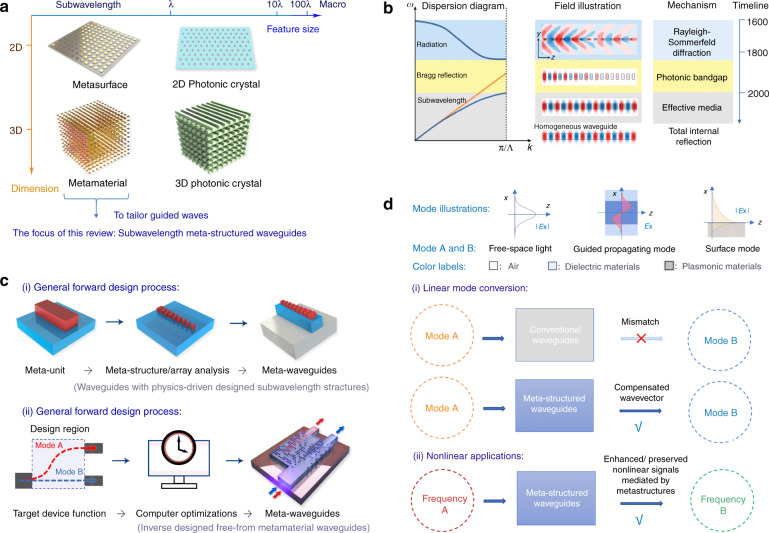
Meta-optics fundamentals and its implementation on waveguides. **a** Concepts of photonic crystals, metamaterials and metasurfaces. **b** Comparisons of light propagating in structured planar waveguides with different periodic feature size^12^. Left panel: schematic dispersion diagram. Right panel: Corresponding electric field distributions at three different regimes, where black rectangles denote silicon segments. (Reproduced from ref. ^12^) Waveguiding mechanisms and rough development timeline are appended^157,537–539^. **c** Two general directions to design meta-waveguides: physics intuition-based forward designs and inverse-design methods^15^. **d** General operation scenarios.

As shown in Fig. 2a, meta-waveguides as the penetration of meta-optics into waveguide optics^51^ can inherit the flexibility and versatility of light manipulation from its free-space counterparts of metasurfaces and metamaterials. The most significant distinction of meta-waveguides from their non-subwavelength equivalents like photonic crystal waveguides is waveguiding mechanism. As is illustrated in Fig. 2b^12^, structures of subwavelength dimension are crucial for meta-waveguides, because in this case the guided electromagnetic waves are experiencing an effective media with designed optical responses^18,19^. This mechanism is physically distinctive from photonic crystals (PhC) that rely on photonic bandgap^140^. Waveguiding in PhC is realized by introducing a line defect supporting guided modes against the photonic bandgap formed by the Bragg-like diffractions from periodic refractive index variations^140^. Taking subwavelength grating waveguides consisting of periodic silicon segments as an instance^12–14^, effective medium theory should be applied. Floquet–Bloch modes are supported without propagation loss^12^ if the period Λ is much smaller than light wavelength *λ*. As Λ gets bigger, light starts to decay as photons are forbidden from propagating within the photonic bandgap. Then the structure will behave as a diffraction grating when the structure period steps into radiation regime^12^.

### General design directions

Ramping up from scattered explorations by applying subwavelength structures^141,142^ and nanoparticles on waveguides^143,144^, meta-waveguides can now be systematically investigated by either forward or inverse-design methods.

In Fig. 2c we plot the two general design process. Except for some cases to elaborate later in next section^83–91,129–134^, most forward-designed meta-waveguides start from the analysis of single meta-atom unit^41–44,48–52,70–76^, which is the most fundamental element to perform field modulation^35,145,146^. By assembling designer meta-units into arrays, a functional meta-structure can be conceived^145,146^ and then integrated with different waveguides. However, this final step requires further attentions to properly engineer phase-matching and mode overlap issues^35,44,51,52^, the specific solutions of which will depend on application and the underlying waveguide platforms. Detailed design methods are elaborated in the following three sections.

As shown in the lower panel of Fig. 2c, inverse-design methods start from a different direction. The target device function is firstly specified in order to determine objective functions. Then computer optimizations are conducted to retrieve device structure under given constraints, such as gradient-based iterations with adjoint methods^15,147^ and deep learning algorithms^148^. Comprehensive design tutorials are detailed in later Section “Inverse-designed metamaterial waveguides”.

Forward-designed meta-waveguides have explicit physical pictures and are relatively straightforward to design with excellent performance. In contrast, inverse-designed metamaterial waveguides are computationally heavy in the implicit optimization process but can realize some sophisticated functionalities that may be hardly assessable by forward design^57,60^.

### General application scenarios

Considering all linear optical devices are mode converters^149^, meta-device functionalities can be ascribed to two general scenarios: linear mode conversion and nonlinear hybrid applications.

In conventional waveguides devoid of structural perturbations, conversion between two arbitrary electromagnetic modes is generally inaccessible due to wavevector mismatch^83,149^. However, in meta-waveguides, the meta-structures can provide an effective momentum **k**_eff_ to enable desired mode conversion^51,82^. The schematic is sketched as Fig. 2d, where Mode **A** and **B** can be free-space light beams, propagating waveguide modes, or surface waves. Lightwave propagating in a meta-waveguide undergoes consecutive and subwavelength-scale delicate field modulations (induced by resonance or form birefringence^35,150–152^) from the engineered structures, leading to an overall wavevector change of the electromagnetic modes^51^. Besides the collective scattering events from the structural perturbations, the mode conversion can be also interpreted as the contribution of multi-path interference from these subwavelength-dimensioned features^153^.

For nonlinear applications, phase-matching is pervasively required owning to energy and momentum conservations. Pronounced nonlinear phenomena usually demand rigorous phase-matched conditions. However, this requirement can be cir-cumvented by integrating meta-structures to waveguides of nonlinear materials to break mode conversion symmetry^52,94,154,155^. Considering the pumping optical mode as TE_00_(*ω*_1_) as an example, the generated nonlinear signal TE_00_(*ω*_2_) are then converted to other modes with same frequency *ω*_2_ but different mode orders: TE_mn_(*ω*_2_) and TE_pq_(*ω*_2_), which will be preserved and accumulated with propagation^52,154^. Back coupling from the retained nonlinear modes to TE_00_(*ω*_2_) is prohibited due to phase mismatch^52^ and minimal field overlap, as the effective wavevector **k**_eff_ provided by the meta-structures is unidirectional^51,82^. In addition, the nonlinear process can also be enhanced by meta-structures with optimized nonlinear overlap^95^.

## Dielectric waveguide-integrated meta-structures

Enabled by the ever-increasing resolution offered by nanofabrication technologies, dielectric meta-waveguides open exciting venues towards versatile chip-integrated applications. This section mainly includes dielectric waveguides with subwavelength surface structures and forward-designed metamaterial waveguides, focusing physical model, design method and applications.

### Tuning scattering properties of nanoantennas on a waveguide

Analogy to well-established microwave and radiofrequency antennas, optical antennas are essential building blocks for manipulating light radiation at subwavelength scale^21–23,156^. Before delving into the details of metasurface-patterned dielec- tric waveguides, we begin with easy interference model of plasmonic optical antennas^40,48–50,94^ and discuss their rational implementations on waveguides for directional coupling applications and so on.

**Fig. 3:**
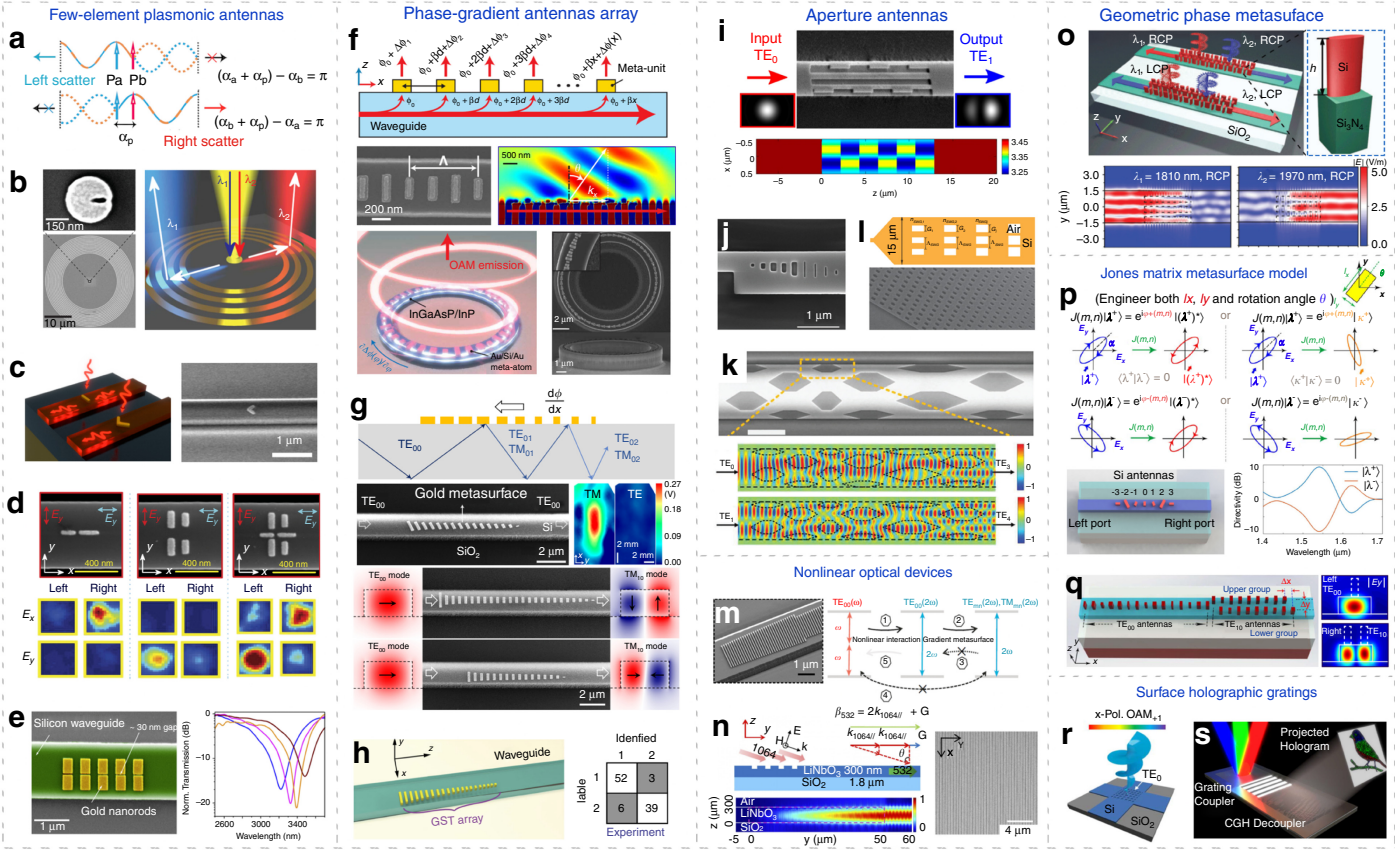
Dielectric meta-waveguides with surface subwavelength structures. **a** Dipole interference model for directional emission and coupling^40,48^. **b** Wavelength-selective demultiplexer by integrating Fano nanoantennas on planar waveguides^49^. **c** On-chip directional coupler^158^. **d** Integrated polarization demultiplexer^50^. **e** On-chip spectroscopic sensor^168^. **f** Guided wave-driven metasurfaces for modulating free-space light^35^. **g** Integrated mode convertor by saddling gradient-metasurface on dielectric waveguides^51^. **h** Programmable metasurface for photonic neural network and experimental results for image recognition^177^. **i**, **j** Waveguide mode convertor based on shallow^84^ or fully etched^87^ metasurface perturbations. **k** Simultaneous multimode convertor^89^. **l** Efficient coupler^38^. **m** Metasurface-assisted second harmonic generation in lithium niobate (LN) waveguide^52^. **n** Nonlinear signal generation and manipulation in LN waveguide patterned with grating metasurface^155^. **o** Polarization and wavelength sorting^42^ for circular polarizations using Geometric phase metasurface. **p** Jones matrix model^43^. **q** Versatile coupler for arbitrary polarizations^44^. **r** OAM generator using subwavelength holographic surface gratings^45^. **s** Multi-color waveguide holography^80^. Panels adapted from: Springer Nature^40,52,177^; ACS^49,84,158,168^; AAAS^45,50,51^; Wiley^38,42,87,88^; OSA^43,44,80,155^.

Starting from the most fundamental model, the response of an optical antenna with dimension much smaller than light wavelength can be approximated by an electric dipole ${{{\mathbf{P}}}}_{{{\mathrm{a}}}} = {{{\mathbf{A}}}}\exp \left[ {{{{\mathrm{i}}}}\left( {{{{\mathbf{k}}}} \cdot {{{\mathbf{r}}}} + \alpha _a} \right)} \right]$ under external electromagnetic stimulus^37,40,48,49^, where **A** denotes radiation amplitude, $\left| {{{\mathbf{k}}}} \right| = 2\pi {{{\mathrm{/}}}}\lambda$ is wavevector, *λ* stands for light wavelength and **r** = (*x*, *y*, *z*) is a spatial vector unit^157^. Acting as a resonator and scatterer, the optical antenna will have a characteristic phase *α*_*a*_ depending on its shape, material, environment index, and light wavelength^21,156^. As is illustrated in Fig. 3a, by applying another antenna dipole ${{{\mathbf{P}}}}_{{{\mathrm{b}}}} = {{{\mathbf{B}}}}\exp \left[ {{{{\mathrm{i}}}}\left( {{{{\mathbf{k}}}} \cdot {{{\mathbf{r}}}} + \alpha _b} \right)} \right]$, directional emission can be achieved^48^ by properly controlling the antennas displacement and initial phase responses *α*_*a*_ and *α*_*b*_. For instance, if we have destructive interference (*α*_*a*_ + *α*_*p*_) − *α*_*b*_ = π in the right side (*α*_*p*_ is the propagation phase determined by antenna displacement)^40^, directional emission to the left side is realized.

Leveraging this intuitive mechanism, antenna combos can be designed on top of dielectric waveguides to realize directional coupling^40,94^. Alternatively, some antenna elements can also appear as aperture, which can be investigated similarly using Babinet’s principle^48^. For example in Fig. 3b, the combination of a gold nanodisk and air nanoslit forms a Fano antenna^49^, which is featured by the asymmetric Fano line-shape originating from the overlap of a broad and narrow resonance from the nanodisk and nanoslit respectively. By saddling this Fano antenna (with opposite bidirectional scattering attributes on two different wavelengths) on rectangular^48^ or planar waveguides^49^, wavelength-selective demultiplexers are realized. In addition to the coherent interference from double-element antennas, directional emission can also take place in a single antenna, when the radiation from different internal plasmonic modes interferes destructively in the other side (Fig. 3c) for directional couplers^158^.

A more general antenna array configuration for high directivity is Yagi–Uda antennas, which are inspired by its radiofrequency counterparts consisting of metal rods acting as feed, reflector and directors^156,159,160^. Endeavors are pursuit for implementing Yagi–Uda antennas to waveguides^37,40,50,160–162^. Waveguide-hybridized gold Yagi–Uda antennas can not only directionally in-couple free-space light source to guided waves, but also out-couple waveguide modes to free space and localized plasmons^37^, with further applications in on-chip data communications, directional lasers, and metrology^160–163^.

Moreover, the scattering attributes of the antenna elements are also polarization-controllable. For instance, the two gold nanorods horizontally resting on a silicon waveguide along the *x* axis (left panel of Fig. 3d)^50^ directionally couple linear *x*-polarization **E**_*x*_ to right-propagating TM mode. However, this array barely interacts with *y*-polarized light **E**_*y*_, as the electrical current in the slender nanorod antenna cannot be efficiently excited when the incident electric field vector is perpendicular to its orientation^21^. In contrast, the structure shown in the middle panel of Fig. 3d can couple incident **E**_*y*_ into left propagating TE mode with high directionality. By combining the two antenna arrays with opposite coupling direction and distinctive response to incident polarizations, chip-integrated polarization demultiplexers for high-speed optical communications are realized^50^.

Plasmonic chains are also incorporated to various dielectric waveguides to explore photon-plasmon interactions, coherent perfect absorptions^164^, coupling^165^, slow light phenomena^166^, all-optical switches^167^ and lab-on-a-chip applications^168–170^, by engineering the interplay between the localized surface plasmon modes and propagating waveguide modes^144,165,166,171,172^. Furthermore, this waveguide-hybrid platform is particularly promising for largely miniaturized spectroscopic and sensing applications^168–170^. Taking the device in Fig. 3e as an instance^168^, a subtle change in surrounding index can be captured by the highly enhanced field in the narrow gap between the gold antennas, resulting in a shift in plasmonic resonance harvested by the waveguide. Compared with conventional bulky free-space optical setups, plasmon resonance can be conveniently measured in this antennas-loaded dielectric waveguides for surface-enhanced infrared absorption spectroscopy, with high coupling efficiency over 70% and compact footprint^168^ around 2 µm^2^.

### Phase-gradient nanoantennas array on waveguide

Besides elementary interference model, next we discuss how to apply metasurface toolbox to design dielectric meta-waveguides for more sophisticated device functions with larger antenna arrays.

#### Guided modes to free-space light applications

Metasurface-decorated dielectric waveguides can efficiently out-couple waveguide signals to complex free-space light fields^79,173^. A guided wave-driven metasurface is schematically shown as the upper panel of Fig. 3f. The total phase shift of the extracted wave from waveguide-fed metasurfaces consists of two parts: (i) the abrupt and spatial-varying phase shift Δ*ϕ*(*x*) provided by each meta-unit^21^ at coordinate *x*, and (ii) phase *βx* accumulated from the propagation of guided waves, where *β* is the propagation constant^35^. Therefore, phase profile of the extracted wave along *x* direction can be formulated as below.

1 $$\phi \left( x \right) = \Delta \phi \left( x \right) + \beta x$$

By judiciously designing the meta-units to tailor Δ*ϕ*(*x*), diverse applications can be realized, such as LiDAR^32,174^, optical communications and display^79–81^. For instance, using the metal-dielectric-metal sandwiched antennas (for approximate 2π phase shift range) to from a phase-gradient metasurface atop of silicon waveguide, off-chip beam deflection and focusing can be achieved^35^. By arranging this metasurfaces on an active InGaAsP/InP microring waveguide to break the degeneracy of clockwise- and counterclockwise-propagating whispering gallery modes (as lower panels in Fig. 3f), a photonic integrated orbital angular momentum (OAM) laser is experimentally demonstrated^35^. In addition to waveguide top surfaces, subwavelength antennas or gratings-like structures can be implemented on waveguide sidewalls as well for structured light and OAM generations^46,175,176^.

#### Guided-mode conversions

As already discussed in the ‘General application scenarios’ section, the meta-structures resting on waveguide can bridge the wavevector mismatch between different modes to enable integrated mode convertors^51^. As is illustrated in Fig. 3g, when guided wave propagates against the unidirectional phase gradient ${{{\mathbf{k}}}}_{{{{\mathrm{eff}}}}} = {{{\mathrm{d}}}}\Phi {{{\mathrm{/d}}}}x$ offered by the collective scattering effect from gradient metasurface, the total internal reflection angle decreases, which corresponds to conversion from low-order to high-order waveguide modes. In contrast, when light propagates along **k**_eff_, its wavevector $k_{{{{\mathrm{mode}}}}} = n_{{{{\mathrm{eff}}}}} \cdot 2\pi {{{\mathrm{/}}}}\lambda$ picks up **k**_eff_, leading to coupling from high-order to low-order modes^51^ (*n*_eff_ denotes effective mode index and *λ* is vacuum light wavelength).

If fundamental modes propagating along **k**_eff_ are injected, it will be coupled into surface waves and get absorbed by the plasmonic metasurfaces. However, light wave with opposite propagating direction can get passed with much lower loss while converting to high-order modes. This asymmetric power transfer facilitates broadband reciprocal optical diodes^82^. Optical neural networks can be also explored utilizing tunable gradient metasurface-based waveguide mode convertors. As shown in Fig. 3h, using Ge_2_Sb_2_Te_5_ (GST) phase-change materials as programmable metasurface waveguide mode convertor, the conversion of two waveguide spatial modes (TE_0_ and TE_1_ modes) can be precisely controlled with 64 distinguishable levels to encode the weight parameters in matrix-vector multiplication computation^177^. A prototypical optical convolutional neural network with 2 × 2 array of the GST convertors is experimentally demonstrated to perform image processing and recognition between handwriting digits “1” and “2”. The experimental recognition results are shown in the right panel of Fig. 3h^177^. Besides, invisibility cloaks and chip-integrated spectrometers can be also envisaged in similar platform^67,93^.

##### Aperture antennas:

The waveguide-integrated plasmonic metasurfaces discussed above inherit Ohmic loss from metals^150^. Next, we discuss meta-waveguides with dielectric subwavelength architectures, which have lower optical loss and better CMOS-compatibility^12,18^. Specifically, the antennas can appear as air apertures defined on waveguides. Either fully- or partially etched aperture antennas can be designed on dielectric waveguides to facilitate mode conversion^83–89^ and efficient coupling applications^38,39^ using effective medium or coupled mode theory^83^.

An exemplary device structure is shown in the upper panel of Fig. 3i, where subwavelength features are partially etched atop of a rectangular silicon waveguide^84^ to create periodic index variations Δ*ε*(*x*, *y*, *z*) along the propagation direction and a graded index profile along the transverse direction. The periodic subwavelength structures along the propagation direction provide an additional momentum **k**_eff_ to enable phase-matched coupling to the desired output mode^83^, while the aperiodic transverse graded index profile enhances coupling strength by optimizing spatial modal overlap^83^. The lower panel of Fig. 3i illustrates the desired refractive index profile offered by the meta-waveguide for converting input TE_00_ mode to TE_10_ mode with high mode purity around 95% and high transmission of 88%^84^.

Besides shallowly etched structures^83–85,88,89^, dielectric waveguides with fully etched subwavelength apertures^86,87,178^ can also enable efficient mode conversions with simplified fabrication process. Figure 3j shows the SEM image of a fully etched meta-waveguide capable of converting TE_00_ mode to TE_10_ mode within an ultrashort length about 2.42 µm around *λ* = 1.55 µm^87^. Integrated multifunctional mode convertors can be also conceived by applying complex surface meta-structures that are also aperiodic in waveguide longitudinal direction^89,178,179^. For instance, the multimode convertor shown in Fig. 3k for simultaneously converting 3 different modes with low insertion loss and acceptable crosstalk^89^. Furthermore, polarization convertors^85^ and high-efficiency optical couplers (Fig. 3l)^38,39^ can be also designed using spatially penetrated waveguides.

#### Nonlinear mode conversions

Metasurfaces can also interface nonlinear mode conversions^180,181^ when incorporating on nonlinear waveguides, as already discussed in Section “General application scenarios”. Figure 3m shows the integrated lithium niobate (LN) waveguide patterned with dielectric gradient metasurface for phase-matching-free second harmonic (SH) generations^52^. As is shown in Fig. 3m, the generated SH signals TE_00_(2*ω*) from the pump TE_00_(*ω*) is coupled to high-order SH modes TE_mn_(2*ω*) and TM_mn_(2*ω*). They are then preserved and strengthened during propagation, as the inverse conversion from high-order SH modes to TE_00_(2*ω*) is prohibited due to phase mismatch^52,154^.

Simultaneous SH generation and radiation are also reported using grating metasurface-patterned LN slab waveguides^155^. By encoding the desired phase and amplitude information to the grating metasurfaces under phase-matching condition (Fig. 3n), the wavefront of the generated SH signals can be efficiently controlled for nonlinear beam-shaping functions like dual focusing and Airy beam generation^155^. Other nonlinear meta-waveguides^94,95,182^ also judiciously optimize nonlinear spatial overlap to significantly enhance nonlinear coupling efficiency^95,183^.

### Jones matrix model for waveguide-integrated metasurfaces

Next, we discuss geometric metasurface-patterned dielectric waveguides and then introduce a more general Jones matrix model for waveguides-integrated metasurfaces.

#### Geometric phase metasurface-on-waveguide

Geometric metasurface or Pancharatnam–Berry phase (PB) metasurface^152,184^ utilizes optical antennas with identical geometry but spatially varying rotation angle *θ* to tailor wavefront of circular polarizations. Meta-units with angular rotation distribution *θ*(*x*, *y*) can locally encode a dispersionless phase profile *φ*^−^(*x*, *y*) to one certain circular polarization |*σ* ^−^〉 and flip its handedness.

2 $$\varphi ^ - \left( {x,y} \right) = 2\theta \left( {x,y} \right)$$

The PB phase shift *φ*^−^ can be traced from the paths of polarization change on the Poincaré sphere (polarization state space)^185^. A PB phase metasurface acting like a half-wave plate will convert incident left-handed circular polarizations (LCP) $\left| {{\it{\upsigma }}^ - } \right\rangle$ to right-handed circular polarization (RCP) $\left| {\sigma ^ + } \right\rangle$ with designer phase modulations^152^: $\left| {\sigma ^ - } \right\rangle \to {{{\mathrm{e}}}}^{{{{\mathrm{i}}}}2\theta \left( {x,y} \right)}\left| {\sigma ^ + } \right\rangle$. In the meantime, the same structure will impart a conjugate phase profile *φ*^+^(*x*, *y*) = −2*θ*(*x*, *y*) to the orthogonal circular polarization $\left| {\sigma ^ + } \right\rangle$ with polarization conversion: $\left| {\sigma ^ + } \right\rangle \to {{{\mathrm{e}}}}^{ - {{{\mathrm{i}}}}2\theta \left( {x,y} \right)}\left| {\sigma ^ - } \right\rangle$. This attribute has been exploited to develop free-space applications such as dual-polarity metalens and holograms^186^.

To transfer geometric metasurface to integrated optics, gold and silicon antennas are patterned on silicon waveguides to realize integrated polarization sorters^41^. The gradually increased antenna rotation angle (with 30° step) creates opposite phase gradient between LCP and RCP light, leading to directional coupling of orthogonal circular polarizations to opposite directions^41^. Figure 3o shows the spin- and wavelength-selective demultiplexers^42^, with simulated coupling efficiency above 50% using silicon antennas-patterned silicon nitride waveguide.

#### Free-space Jones matrix model for metasurface

Despite the simple relationship between imparted phase *φ* and antenna rotation angle *θ* for convenient design, meta-waveguides discussed above inherit limitations from geometric metasurface. First, the design methods are only applicable for circular polarizations. Second, the optical fields that coupled into the waveguide are hybrid modes^41,42^, which impedes high-speed optical communications applications due to inter-mode dispersion. Another important category of metasurface exploring propagation phase, where antenna orientations *θ* are fixed but the width *l*_*x*_ and height *l*_*y*_ of each antenna cell are individually engineered to tailor the eigen-phases *φ*_*x*_ and *φ*_*y*_ encoded to two orthogonal linear polarizations^152^: $\left| {{{\boldsymbol{x}}}} \right\rangle \to {{{\mathrm{e}}}}^{{{{\mathrm{i}}}}\varphi _x}\left| {{{\boldsymbol{x}}}} \right\rangle$ and $\left| {{{\boldsymbol{y}}}} \right\rangle \to {{{\mathrm{e}}}}^{{{{\mathrm{i}}}}\varphi _y}\left| {{{\boldsymbol{y}}}} \right\rangle$. Propagation phase stems from form birefringence^43,151^, as the dielectric antennas acting as truncated small waveguides have different effective mode indices under different incident polarizations. This leads to different accumulated phase retardations when light passing through the antennas with different geometry.

Jones matrix metasurface model combines both geometric phase and propagation phase^151,152^. A periodic metasurface resembling a birefringent waveplate can be described by a Jones matrix **J** (connecting two input and output polarization vectors as ${{{\mathbf{J}}}}\left| \lambda \right\rangle = \left| \kappa \right\rangle$ with two operation scenarios, as illustrated in Fig. 3p:

(i) Impart two independent and arbitrary phase profiles *φ*^+^(*m*, *n*) and *φ*^−^(*m*, *n*) to an arbitrary pair of orthogonal elliptical
  polarizations $\left| {\lambda ^ + } \right\rangle$ and $\left| {\lambda ^ - } \right\rangle$ respectively^151,152^, where (*m*, *n*) denotes different antenna pixels^43^. The unitary and symmetric matrix nature of **J** guarantees the following two mappings simultaneously^152^,
  3 $${{{\mathbf{J}}}}\left( {m,n} \right)\left| {{{{\mathbf{\lambda }}}}^ {+} \rangle = {{{\mathrm{e}}}}^{{{{\mathrm{i}}}}\varphi ^ + \left( {m,n} \right)}} \right|\left( {{{{\mathbf{\lambda }}}}^ + } \right)^ \ast \rangle \,{{{\text{ and }}}}\,{{{\mathbf{J}}}}\left( {m,n} \right)\left| {{{{\mathbf{\lambda }}}}^{-} \rangle= {{{\mathrm{e}}}}^{{{{\mathrm{i}}}}\varphi ^ - \left( {m,n} \right)}} \right|\left( {{{{\mathbf{\lambda }}}}^ - } \right)^ \ast \rangle$$
  where superscript * represents complex conjugate. Here, the two independent phase profiles *φ*^+^(*m*, *n*) and *φ*^−^(*m*, *n*) can be arbitrarily assigned, but the output polarizations $\left| {\kappa ^ + } \right\rangle$ and $\left| {\kappa ^ - } \right\rangle$ are fixed (as left panels of Fig. 3p): $\left| {\kappa ^ + } \right\rangle = \left| {\left( {\lambda ^ + } \right)^ \ast } \right\rangle$ and $\left| {{{{\mathbf{\kappa }}}}^ - } \right\rangle = \left| {\left( {{{{\mathbf{\lambda }}}}^ - } \right)^ \ast } \right\rangle$, denoting preserved polarization ellipse but flipped handedness. The desired Jones matrix **J**(*m*, *n*) can thus be solved to determine antenna structure^151^.
(ii) Complete control over both the phase *φ*^+^(*m*, *n*) and output polarization $\left| {\kappa ^ + } \right\rangle$ of one certain incident polarization $\left| {\lambda ^ + } \right\rangle$, while the encoded phase *φ*^−^(*m*, *n*) for its orthogonal polarization $\left| {\lambda ^ - } \right\rangle$ is not configurable^151^ (right panels of Fig. 3p).

#### Implementation on waveguide

Integrating the Jones matrix metasurface model on dielectric waveguides can propel integrated optics into new heights by offering a more general design method for versatile multiplexers for arbitrary polarizations and complete mode control over coupled lights. By assigning opposite phase gradients to two orthogonal incident polarizations $\left| {\lambda ^ + } \right\rangle$ and $\left| {\lambda ^ - } \right\rangle$ under operation scenario (i), integrated polarization demultiplexers can be devised with high directivity over 20 dB for arbitrary incident elliptical polarizations^44^ (lower panel of Fig. 3p). Furthermore, by optimizing antenna geometries at multiple wavelengths^43,44^, dispersion-managed wavelength demultiplexers or ultrabroadband directional couplers can be also conceived.

Notably, when transferring dielectric meta-units designed by Jones matrix method^151,152^ to waveguides, spatial modal overlap between antenna scattering nearfield and target waveguide mode for excitation requires further attention^51^. For instance, to selectively excite TE_m,n_ mode, *m* + 1 rows of antennas are required^44,187^. The mode index *n* is then controlled by properly engineered phase-gradient Δ*φ*/*d* of the metasurface by combining generalized Snell’s law with phase-matching condition^21^,

4 $$\left| {\Delta \varphi {{{\mathrm{/}}}}d} \right| = n_{{{{\mathrm{eff}}}}} \cdot k_0$$

where Δ*φ* and *d* are the phase difference and distance between adjacent antennas respectively, *n*_eff_ represents the effective index of the target waveguide mode to excite, *k*_0_ is wavevector. As depicted in Fig. 3q, specific high-order mode of interest can be exclusively launched by the mode-configurable coupler with high purity^187^ over 90% under operation scenario (ii). On-chip OAM generators with configurable topological charge is also proposed using mode mixing method^44,187^.

### Holographic gratings and hybrid subwavelength surface structures

For chip-scale structured light generations, subwavelength holographic gratings are applied on top of silicon waveguides^45,77,78,188,189^ to generate surface-emitting vortex beams carrying OAM with specific topological charge^77^ $\ell$. To design such device, the holographic grating can be retrieved by interfering the target OAM mode ${{{\mathbf{E}}}}_{{{{\mathrm{OAM}}}}} = {{{\mathbf{A}}}} \cdot \exp \left( {{{{\mathrm{i}}}}\ell \theta } \right)$ and approximated in-plane waveguide field ${{{\mathbf{E}}}}_{{{{\mathrm{waveguide}}}}} = {{{\mathbf{B}}}} \cdot \exp \left( {{{{\mathrm{i}}}}k_{{{{\mathrm{mode}}}}} \cdot x} \right)$ as^45^

5 $${{{\mathrm{G}}}}_{{{{\mathrm{fork}}}}} = \left| {{{{\mathbf{E}}}}_{{{{\mathrm{OAM}}}}} + {{{\mathbf{E}}}}_{{{{\mathrm{waveguide}}}}}} \right|^2$$

where **A** and **B** are amplitudes, *θ* is azimuthal angle and *k*_mode_ denotes the propagation constant of the waveguide mode propagating along *x* direction. Figure 3r shows the proposed broadband OAM generator using two superposed subwavelength holographic forks atop of a silicon waveguide with a compact footprint^45^ of 3.6 × 3.6 µm^2^, where G_fork_ is converted to binary phase hologram considering current fabrication conditions. Similar subwavelength holographic surface architectures are leveraged to realize waveguide-integrated holography as well (Fig. 3s)^80,81^.

Besides, inspired from the integrated trench metalens and one-dimensional transmit-array^190^, on-chip wavefront shaping and mathematical optical signal transformations are demonstrated using standard silicon-on-insulator (SOI) platform with subwavelength metalens apertures^53,191,192^. Metamaterials are also saddled on waveguides for various integrated photonics applications such as optical modulation, coupling and sensing^193,194^.

### Subwavelength grating waveguides

**Fig. 4:**
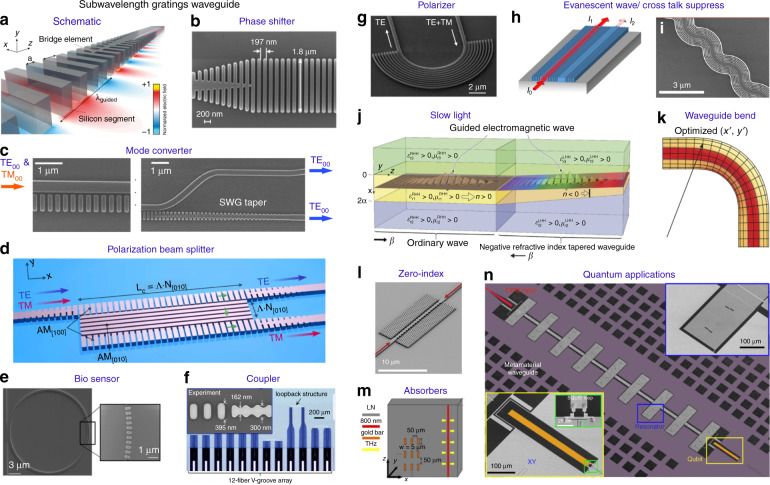
Forward-designed metamaterial waveguides. **a** Schematics of a subwavelength grating waveguide^12^. **b** Nanophotonic phase shifter^56^. **c** Polarization mode convertor^196^. **d** Broadband polarization beam splitter^198^. **e** SWG ring resonator biosensor^90^. **f** Integrated fiber-to-chip coupling interfaces. Inset: details of the SWG waveguide coupler^203^. **g** Ultrabroadband chip-integrated all-silicon polarizer^205^. **h**, **i** Silicon metamaterial waveguides for exceptional coupling^55^ and evanescent wave suppression^54^. **j** Left-handed metamaterial waveguiding heterostructures to slow light^131^. **k** Schematic of waveguide bend transformation^135^. **l** Integrated zero-index waveguide^216^. **m** Terahertz band-stop filer using a metamaterial slab waveguide^219^. **n** Metamaterial waveguide for qubits. Insets: SEM images for the capacitive qubit coupler (lower panel) and coupled microwave resonators respectively (upper panel)^225^. Panels adapted from: Springer Nature (**a**^12^, **i**^54^, **j**^131^, **k**^135^, **n**^225^); OSA (**b**^56^, **e**^90^, **g**^205^, **h**^55^, **m**^219^); IEEE (**c**^196^, **f**^203^); Wiley (**d**)^198^; ACS (**l**)^216^.

The meta-waveguides discussed in previous subsections are featured by subwavelength patterns on waveguide top surfaces, as summarized by Fig. 3. In the following two subsections, we focus forward-designed metamaterial waveguides (see Fig. 4), which are devised mainly by effective medium theory or transformation optics^16–19^.

Among them, subwavelength grating (SWG) waveguides exhibit promising potentiality to revolutionize conventional photonic integrated circuits^12^. Given that this topic is already reviewed in previous literatures^12–14^, here SWG waveguides as a specific member of dielectric meta-waveguides family are only very briefly mentioned to keep this review intact.

A typical structure for SWG waveguide is illustrated as Fig. 4a, where periodic silicon segments with subwavelength spacing Λ forms the waveguide core^12^. When is Λ much smaller than the half-wavelength of the guided light, localized Floquet- Bloch modes are supported without scattering loss in propagation^14^. Distinctive from Bragg and conventional diffraction gratings, the SWG structure instead behaving as a homogeneous uniaxial crystal^195^ controlled by grating structures and duty cycle. ‘Bridge’ elements are commonly applied for the low-loss transition between SWG and homogeneous waveguide parts.

Compact and broadband passive phase shifters^56^ and mode convertors^196,197^ are demonstrated as Fig. 4b, c respectively, via engineering the dispersion and anisotropy of the SWG metamaterial waveguides. Polarization beam split- ters^198–201^, polarizers^202^, SWG microring-based biosensors^90,91^ and high-efficiency fiber-to-chip optical couplers^203,204^ are also explored, with typical device structures shown in Fig. 4d–f.

### Other forward-designed metamaterial waveguides

#### Integrated photonics applications

Figure 4g shows the SEM image of an on-chip silicon polarizer with broad bandwidth exceeding 415 nm and high polarization extinction ratio over 20 dB at telecommunication band^205^, using a 180°-bend silicon waveguide coupled with SWG metamaterial claddings. Moreover, SWG ‘claddings’ can be exploited to realize exception coupling and suppress evanescent wave as well (see Fig. 4h, i)^54,55^, for higher integration density and crosstalk mitigation. Optical beams focusing and transformations for guided waves signals are also reported^206,207^.

#### Exotic optical physics

Metamaterial waveguides are ideal platforms for exploring exotic physical phenomena to theoretically enable light slowing and trapping^131^, such as the axially varying waveguiding heterostructure with a metamaterial core in negative refractive index (Fig. 4j). Although the pace of photons may not be stopped in realistic metamaterial waveguides with appreciable bandwidth^132^, abundant optical physics for light-matter interactions can still be explored, with potential applications in chip-integrated optical signal processing and communications^133,208^.

Leveraging transformation optics (TO)^19,209^, integrated metamaterial waveguides can be conceived^129,135,210–213^. Figure 4k illustrates the schematic for designing a low-crosstalk multimode waveguide bend with feasible fabrication constraints using TO^135^, where darker color indicates higher index. Compared with conventional bending waveguides, significant inter-mode coupling mitigation (over 14 dB) in this TO waveguide is experimentally verified^135^. Waveguide tapers, connectors and beam expanders are also designed under TO theory^129,210,211^.

Moreover, metamaterial waveguides with exotic refractive indices are also an active field of research^214^. For instance, theoretical perfect waveguide bending can be realized in zero-index metamaterials^134,215^. Figure 4l illustrates the on-chip metamaterial waveguide based on standard SOI platform with a refractive index approaching zero^216^, where phase-matching free light propagation is directly observed. Other exotic optical phenomena are also explored at optical and lower frequencies, such as strong field enhancements in epsilon-near-zero materials^217,218^, magnified light absorption (Fig. 4m)^219,220^, enhanced spin Hall effect and broadband mode conversions^130,221–223^, to name a few.

#### Quantum applications

Recently, quantum supremacy is demonstrated using programmable superconducting processors for qubits^224^, where the computation time can be dramatically accelerated from over millenniums to minutes compared with classical processors under certain calculation scenarios. In the meantime, computational advantages using quantum photonics are also reported^6^. In contrast to conventional structures, metamaterial waveguides can enable new degrees of freedom in controlling qubits^225–227^. Figure 4n shows a superconducting metamaterial waveguides to tailor the transition lifetimes of qubits^225^, where metastable qubit states with ultralong lifetime (about 24-fold enhancement) and short-lived states are observed and selectively tuned.

## Optical meta-fibers

Optical fibers are another well-established platform to guide propagating electromagnetic modes. Integrating meta-structures with fibers gives rise to optical meta-fibers as an important member of meta-waveguide family. Distinctive from dielectric nanophotonic waveguides for various on-chip applications, optical fibers are widely used for massive long-haul optical communications and versatile interconnects that interface chips to exterior systems^2,3^. Moreover, their flexibility, biocompatibility, and mechanical robustness have made optical fibers with small cross-section and extreme aspect ratio as excellent candidates for remote, in situ and in vivo applications^228,229^ beyond photonic integrated circuits, such as biochemical sensors and endoscopic optical imaging^230^.

However, conventional optical fibers are limited by silica material properties (in terms of transparency window, cut-off frequency and nonlinearity) and the cylindrical waveguide geometry pervasive in fiber drawling process^229^. Compared with microfibers devoid of subwavelength features^230^, optical fibers employing subwavelength meta-structures allow for much powerful control over light attributes, including phase, polarization, amplitude, dispersion, and optical impedance^229^. A novel technological roadmap (lab-on-fiber) was thus launched to develop a novel class of all fiber devices and components, by judiciously integrating functional structures and materials onto optical fiber substrates at the micro- and nano-scale^105,231–233^. This vison is expected to provide the foundational basis to enlarge the functionalities pertaining to optical fiber technology towards ‘plug-and-play’ platforms to be exploited in many strategic applications, ranging from optical processing and computing to environmental monitoring, life science, safety and security^75,234^.

In general, meta-structures can be designed on the flat tip of optical fibers^74–76,96–102,232,235^ to enable ‘meta-tips’, where the fiber facet acts as a unique light-coupled microscopic substrate. Alternatively, subwavelength architectures can be also devised along the fibers for exotic waveguiding or to interact with evanescent optical fields to enable metamaterial fibers^236–247^.

In this section we discuss optical fiber ‘meta-tips’ (fiber facet-integrated meta-structures) and ‘meta-fibers’ (intra-fiber subwavelength features), showcasing representative applications and corresponding fiber fabrication technologies.

### Optical fiber meta-tips

**Fig. 5:**
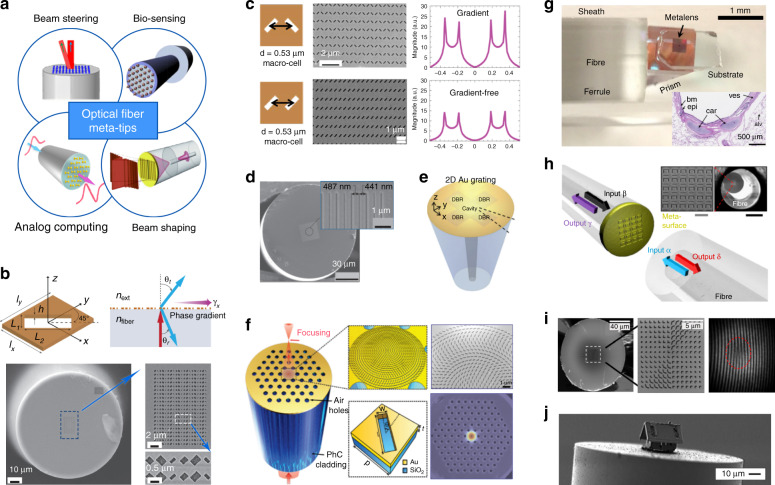
Optical fiber meta-tips. **a** Combining the advantages of optical fibers and the functionality of metasurfaces for novel devices. Insets adapted from: Elsevier (Bio-sensing^255^); AIP publishing (Beam shaping^256^). **b** Schematics and SEM images of an optical fiber meta-tip for beam steering^75^. **c** Upper panels: Metasurface-enhanced lab-on-fiber bio sensors. Lower panels: Gradient-free benchmark^76^. **d** SEM images of the fiber facet-integrated metamaterial dispersive gratings^107^. **e** Multiparameter nanoprobe using a plasmonic crystal cavity on fiber end-face^254^. **f** Metalens on photonic crystal (PhC) fiber facet: schematics and SEM images of the meta-structures^100^. **g** Photograph of the nano-optic endoscope using metalens^74^. Inset: In vivo endoscopic imaging. **h** Fiber-optic meta-device for all-optical signal modulation^96^. **i** SEM image of meta-facet fiber to generate OAM_+1_ with measured tilt interferogram^257^. **j** SEM image of a tiny microhouse assembled on a fiber tip^260^. Figures adapted from: Springer Nature (**b**^75^, **e**^254^, **g**^74^, **h**^96^); Wiley (**c**)^76^; AIP (**d**^107^, **i**^257^, **j**^260^); De Gruyter (**f**)^100^.

As highlighted in past review papers^22,229^, the judicious integration of metasurfaces to optical fibers could be a game-changing direction for next-generation fiber-optic devices and components to disruptively expand their conventional functionalities. As is illustrated in Fig. 5a, metasurfaces would, indeed, open unprecedented paths to manipulate light using optical fibers to significantly accelerate relevant developments such as active beam profilers, spatial light modulators and fiber-optic tweezers, just to name a few^231^. Moreover, translating flat optics onto unconventional substrates, as the case of optical fibers, would allow the creation of lab-on-fiber assisted platforms with extraordinary capabilities in biomedical imaging, scanning near-field optical microscopy, and single-molecule detection^103,106–110,248–253^.

Following this intriguing suggestion, one of the important milestones as ‘optical fiber meta-tip’ was first demonstrated by Principe et al.^75^, with extraordinary light manipulation capability. The proof-of-concept was realized via focused-ion-beam (FIB) milling of rectangular nanoantennas arrays on the gold-coated termination of a single-mode optical fiber. The metasurface was designed to display a linear-phase profile to split an impinging light beam into an ordinary polarized component, and an anomalous extraordinary component (with different polarization state) for beam steering, as shown in Fig. 5b. By judiciously designing phase-gradient metasurface on fiber tips, the same group later reported the efficient excitation of plasmonic surface waves with unprecedented localization levels for developing all fiber optrodes with unparalleled sensing capability for life science applications (Fig. 5c)^76^. A phase-gradient plasmonic metasurface-based device was demonstrated to detect biomolecular interactions, with a sensitivity more than two orders of magnitude higher than that of a gradient-free counterpart.

The alluring potential of integrating meta-structures on optical fibers are soon recognized by researchers, followed by massive emerging optical fiber meta-components and meta-devices for various applications. As shown in Fig. 5d, a high- quality metamaterial dispersive gratings is integrated on fiber facet with the quality factor exceeding 300 to efficiently tailor light dispersion^107^. Lab-on-fiber nanoprobes using in plane integrated distributed Bragg reflectors on the end-face of a single-mode optical fiber is also reported, enabling the excitation of two spatially separated high-Q resonance modes (Fig. 5e)^254^. Figure 5f shows the first focusing optical meta-tip made by directly patterning a gold meta-lens on the facet of a photonic crystal fiber^100^. Superfocusing is achieved at 1550 nm wavelength with maximum enhanced optical intensities reaching 234%.

This concept could also have an enormous impact on next-generation fiber-optic imaging systems, especially in life science applications^231^, with exceptional spatial resolutions and miniaturization levels^255^. The reliable integration of metalenses could considerably overcome traditional difficulties associated with optical aberrations, alleviating also the well-known trade-off between the transverse spatial resolution and depth of focus, which significantly limits the scope of optical imaging in precision medicine. Optical fiber meta-tips coupled with optical coherence tomography (OCT) tools may provide the key asset for advanced nano-endoscopes, for in vivo imaging with spatial resolutions that are hardly possible with conventional platforms. Figure 5g shows a novel matalens-assisted endoscopic OCT platform that can be easily integrated in needles and catheters to achieve near-diffraction-limited imaging through negating non-chromatic aberrations^74^. The proposed tool was validated in case of endoscopic imaging of human lung specimens and sheep airways (shown in Fig. 5g inset), demonstrating superiority as compared to commercial OCT endoscopes featuring to improve and enlarge the clinical utility of OCT platforms.

The extraordinary capability of metasurfaces to control light at nanoscale would also open potential disruptive developments in optical signal processing applications. As illustrated in Fig. 5h, coherently controlled absorption in a fully fiberized and packaged switching meta-device was recently reported^96^. An optical fiber meta-tip platform was conceived demonstrating logical functions (XOR, NOT and AND) at wavelengths between 1530 and 1565 nm. The proposed metadevice has been successfully tested at up to 40 gigabits per second, opening new opportunities for 100 THz bandwidth single-photon operation with potential impact in quantum information networks^97,98^. Meanwhile, fiber facet-integrated meta-structures are also exploited for structured light generations and beam shaping^256–259^. Figure 5i sketches the SEM images of the meta-facet fiber for generating OAM beam with topological charge $\ell = + 1$^257^.

These valuable examples highlight future potentials of lab-on-fiber technology, optical communications, and related optical fiber meta-tips to drive a new technological revolution in optical fiber technology, for many realistic applications with unrivaled advantages in terms of functionalities, miniaturization, power consumption and overall performances. Next development in this domain may also take advantage from the advances in integrating functional materials onto optical fibers with a full spatial control at nanoscale. It is now possible to conceive and create any arbitrary 2D and 3D micro and nanostructure on the facet of optical fibers, as demonstrated by the smallest micro-house never realized on the optical fiber termination using robotics nano-factory and origami techniques^260^ (see Fig. 5j).

### Metamaterial fibers

**Fig. 6:**
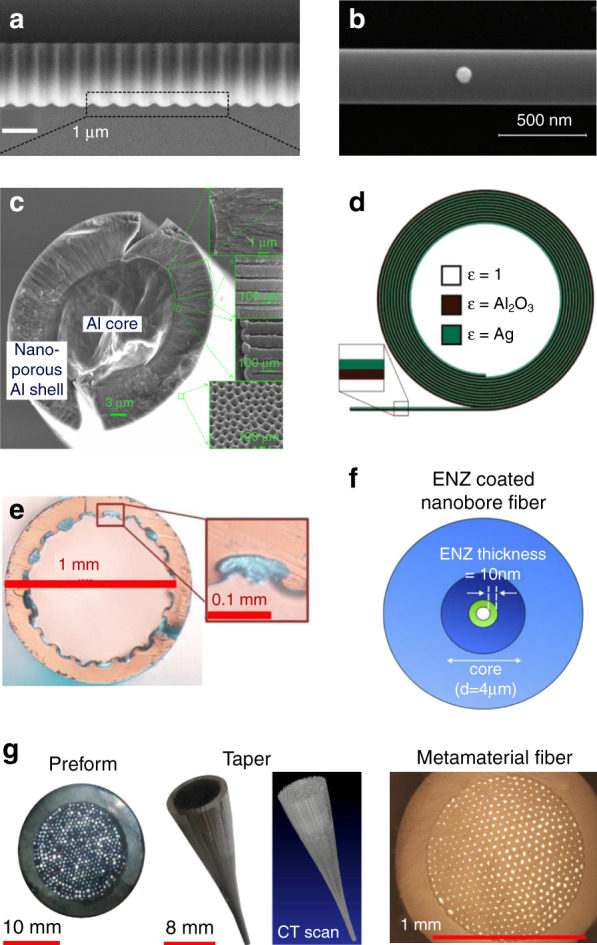
Optical meta-fibers. **a** Fiber gratings with subwavelength feature size^236^. **b** Air-clad silica nanofiber with a gold nanoparticle resting on its surface^238^. **c** SEM image of the metamaterial fiber with aluminum core and radially oriented nanopores^274^. **d** Cross-section schematic of a rolled-up Al_2_O_3_/Ag bilayers hollow fiber^240^. **e** Microscope image of the fiber facet with subwavelength indium wires uniformly embedded in Zeonex host around the hollow-core^243^. **f** Hollow-core ENZ fiber embedded with ITO nano-shell^245^. **g** Metamaterial fiber for subwavelength-resolved THz focusing and imaging^246^. Panels adapted from: OSA (**a**^236^, **c**^274^, **e**^243^); AAAS (**b**)^238^; ACS (**d**)^240^; Springer Nature (**f**^245^, **g**^246^).

Innovations can be also made beyond fiber facets. Compared with optical meta-tips, metamaterial fibers provide a broader stage to deploy meta-structures to tailor the propagation and dispersion of fiber modes, yet they also demand more stringent quality control over the desired subwavelength structures along the entire fiber^229^. For meta-fibers with complex sidewall structures, Fig. 6a shows the SEM image of a microfiber Bragg gratings fabricated by FIB milling working around 1.55 µm communication wavelengths, with 576 nm pitch and 100 nm-depth grooves^236^. This fiber device with subwavelength features exhibit high transmission dip around 15 dB^236^ and high sensitivity for refractive index and temperature^237,261–263^. Simple nanostructures like a single gold sphere can be also applied to airlclad nanofibers to explore spin-orbit interaction of light and controlled directional coupling^238^ (Fig. 6b). Intra-fiber metasurfaces are proposed for mode conversion^239^. Other sophisticated fiber sidewall textures may be envisaged by adapting emerging fiber fabrication technologies to optical fibers^264–266^.

Subwavelength structures that are uniform along fiber length direction can be manufactured by preform-based fiber drawing methods (detailed in next subsection)^267–269^. Metamaterial fibers^244,268–271^ with hollow^240–243,272,273^, metallic^274,275^, holey^276^ and multiple cores^246,247,276,277^ are reported. Figure 6c shows the cross-section of an anisotropic metamaterial fiber with radially distributed internal nanopores around the aluminum core supporting exotic modes^274^ to strongly modify the waveguiding fiber attributes. For instance, hollow-core fibers with hyperbolic metamaterial claddings can guide light beyond cut-off frequency for enhanced light coupling^278^ and low latency communications. Metamaterial cladding with various material combinations enables powerful dispersion control from ultraviolet^240^, infrared^241,242^ to terahertz (THz)^243,278^ and microwave frequencies^279^ to circumvent the structure and material property limits of conventional silica optical fibers^280^. The meta-fiber shown in Fig. 6d exploits surface plasmon and classical fiber waveguiding^240^. Figure 6e sketches a single-mode, single-polarization hollow-core fiber with metal-dielectric hybrid metamaterial cladding for high-density integration of THz systems^243^.

Epsilon-near-zero (ENZ) materials such as indium tin oxide (ITO)-embedded hollow step index fiber is also proposed as Fig. 6f^245^, with strong field enhancement in the subwavelength ITO shell and potential applications in sensing, nonlinear optics and enhanced quantum emissions^281^. Figure 6g depicts the cross-section of a THz metamaterial fiber with hexagonally arranged subwavelength indium wires for sub-diffraction imaging^246^ produced by fiber drawing of the preform. This metamaterial fiber inspired by hyperlenses with indefinite permittivity tensor can collect high spatial frequencies over optically long distances to enhance imaging resolution from THz^277^ to infrared frequencies^282^.

### Fabrication technologies

**Fig. 7:**
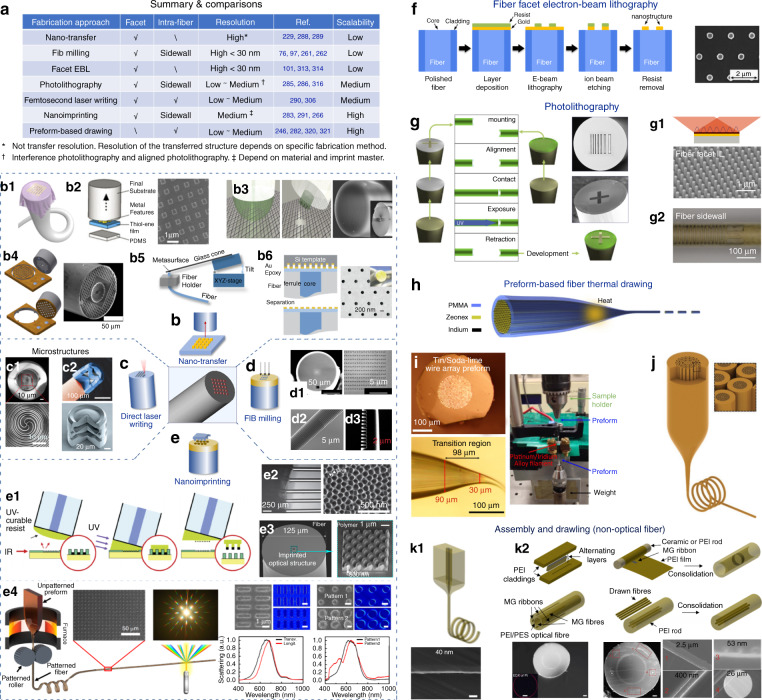
Fabrication technologies for meta-structured fibers. **a** Summary of different fiber fabrication methods^76,229,283,287–294^. **b** Nanotransfer. **b**1 Wet-transferred gold nanoslit grating membrane on a fiber facet^288^. **b**2 ‘Decal transfer’ schematics^296^ and SEM image of the transferred structure. **b**3 Template stripping transfer via UV-curable polymer (green hemisphere) on fiber tip^289^. **b**4 Mounting technique^103^. **b**5 Micromanipulation set-up^299^. **b**6 Template transfer method and the fabricated fiber facet^301^. **c** Femtosecond laser writing lithography. **c**1 Fiber facet-integrated spiral zone plate^290^. **c**2 On-fiber multi-lens objectives and structure details^305^. **d** FIB milling. **d**1 Fabricated plasmonic fiber meta-tip^76^. **d**2 SEM image of a tapered fiber probe grating^261^. **d**3 Fiber tip engraved by FIB^262^. **e** Nanoimprint. **e**1 Nanoimprint and transfer lithography procedures^310^. **e**2 SEM images of U-grooves for fiber array alignment and the imprinted structures^291^. **e**3 The imprinted fiber facet and close-up structure details^311^. **e**4 Direct imprinting thermal drawing schematic and resolution test patterns (**e**5)^266^. **f** Process schematic for EBL on fiber facet and SEM image of the fabricated structure^101^. **g** ‘Align-and-shine’ photolithography^286^. Insets: fabricated structures. **g**1 Interference lithography (IL). Lower panel: Fabricated patterns using IL^285^. **g**2 Resist patterns produced by 3D photolithography^316^. **h** Thermal drawling of a metamaterial fiber^246^. **i** Metamaterial fiber preform and a typical drawling transition region^282^. **j** Iterative drawling with assembled fibers array^320^ and (**k**1) single fiber obtained from previous steps^321^. (**k**2) Preform assembly schematic to produce various metamaterials fibers (with SEM images shown in lower panels)^321^. Panels adapted from: ACS^101,103,288,296^; IEEE^262,289^; OSA^261,282,285,299^; The Royal Society^301^; AIP Publishing^290,310^; Springer Nature^246,266,305,320,321^; Wiley^76,291^; IOP Publishing^286,311,316^.

The waveguiding flexibility, high aspect ratio and microscopic cross-section of optical meta-fibers provide unique advantages, but simultaneously challenge conventional nanofabrication technologies developed for large planner substrates^228^. Nevertheless, the maturity of diverse fiber fabrication techniques has made optical fiber meta-tips and metamaterial fibers one step closer from lab demonstrations to potential market products. Figure 7a summarizes currently available approaches to fabricate meta-structured optical fibers^76,97,104,228,229,283–292^. For optical meta-tips, ordinary photo- and electron beam lithography generally confront challenges in sample mounting and uniform resist coating for the small fiber facet^229,287^. Instead, nanotransfer^103,110,229,288,289^ and FIB milling^75,76,96^ can be applied to define exquisite subwavelength meta-patterns at fiber facets. Laser writing^290,293^ and nanoimprinting^283,284,291,294^ generally have comparatively lower resolution but higher yield. For metamaterial fibers, preform-based fiber drawing methods are most promising for mass-production. Other approaches such as FIB milling and nanoimprinting can produce subwavelength architectures at fiber sidewalls^236,237,266^. Different fabrication technologies for meta-structured fibers are briefly compared as the following.

#### Nano-transfer

Nanotransfer is a straightforward method that transports as-fabricated meta-structures to fiber facets to circumvent the difficulty of direct nanofabrication on small fiber tips, as shown in Fig. 7b. The transferred meta-structures inherit the high resolution from conventional nanofabrication methods. However, most transfer processes have relatively low throughput and defects can be induced during the transfer. Figure 7b1 illustrates a wet-transferred meta-tip working as a flexible passband filter^288^ using sacrificial layer and water bath scooping. The meta-structures can be also fabricated by ‘nanoskiving’ techniques in an inexpensive manner^229,295^ for wet-transferred to fiber facets. For dry transfer, a ‘decal transfer’ technique using sacrificial films (Fig. 7b2)^296,297^, and a cost-effective template stripping method using ultraviolet (UV) light-curable hybrid polymer (Fig. 7b3)^289^ are proposed. Another ‘stamping-like’ method is sketched as Fig. 7b4^103^, where the plasmonic metasurface is mounted at fiber facet via epoxy^298^. Direct mechanical transfer is also reported^299^ (Fig. 7b5). Figure 7b6 depicts a template transfer approach^300^ to produce high quality and robust plasmonic structures for real-time biosensing^110,301^.

#### Direct laser writing

Leveraging femtosecond laser pulses and two-photon polymerization^302,303^, direct laser writing can be applied to define micro- structures at fiber facets. Despite submicrometer resolution has been achieved by this multiphoton lithography technology^302,304^, currently demonstrated structures on fiber facets generally have feature sizes bigger than light wavelength^290,292,293,305–307^. Nevertheless, fiber tips-integrated subwavelength meta-structures can still be envisaged with the steady trend of resolution improvement. Fig. 7c1 shows the SEM image of the laser-written micro-spiral zone plate at fiber facet for OAM generation^290^.

Although currently available material library for two-photon polymerization is slightly restrained, this approach, more importantly, can fabricate three-dimensional (3D) complex structures that are hardly accessible by conventional top-down lithography^292,305^. Polymerization only takes place in the vicinity of the laser focal spot and the location of the solidified voxels can thus be manipulated much freely^302^. Beside fiber facets, intra- waveguide sophisticated structures may be also defined by this technology^308,309^. Optomechanical^307^ and bionic structures^303^ can be fabricated as well. Figure 7c2 shows the colored SEM image of a compact triplet lens objective attached to an optical fiber^305^ for miniaturized endoscopes and imaging applications.

#### Focused-ion beam milling

FIB milling is another technology to produce intricate 3D nanostructures with high fabrication resolution. Precise subwavelength features can be defined at fiber facets^75,76,96^ and fiber sidewall^236,237,261–263,266^. Figure 7d1 shows the SEM image of an optical fiber meta-tip^76^, where a thin gold layer is first deposited on fiber facet and the metasurfaces are then written by FIB milling. Besides, elegant fiber sidewall architectures are also demonstrated (see Fig. 7d2, d3)^261,262^. Albeit to the advantages in fabrication accuracy and 3D nanostructuring, FIB has a slow throughput that precludes mass production. The optical response of the engraved nanostructures may be influenced by the gallium ions doping during the process^229^.

#### Nanoimprinting

Nanoimprint lithography is a high-throughput and cost-effective technology that utilizes molds to replicate predefined stamp topographies^138^. This method can produce various nanopatterns on fiber facets^284,291,294,310–312^ and fiber sidewall in parallel^266^. Figure 7e1 illustrates a nanoimprint process to transfer subwavelength period metal grating from the mold to fiber facet^310^ with a sub-15 nm feature using PDMS and SU-8 for chip-scale probing and testing, where U-grooves are optimized to settle the alignment issue^291^. However, for complex metasurface-on-fiber structures, the imprinting and alignment accuracy may still need amelioration. 3D nanostructures are also accessible^284,311^, such as the 3D beam splitter shown in Fig. 7e3^311^. Meanwhile, when combined with fiber drawling, nanoimprinting may also produce surface structures at fiber sidewall using roller molds (Fig. 7e4)^266^. Figure 7e5 depicts the fabricated patterns for resolution test. For its potential applications to optical fibers, the imprinted nanostructures have to stay close to the fiber core in order to evanescently interfere with the optical modes.

#### Electron beam and photolithography

Electron-beam lithography (EBL) is a time-consuming process but can produce high-quality subwavelength structures with high resolution. To apply EBL to define patterns on the tiny facets of optical fibers, modifications on apparatus are quired^313^, as conventional spin coating and exposure platforms are designed for big wafers. Figure 7f illustrate the exemplary procedures to pattern on facets using EBL (left panels), and the SEM image of the fabricated structures after ion-beam etching (right panel)^101^. ‘Dip and vibration’ coating technique^314^ is proposed for better resist coating uniformity. Customized rotating chuck and exposure holders are also explored to obtain better lithographic patterns on fiber facets^313^.

Photolithography has higher yield when working for large substrates, yet it also encounters challenges on resist coating, sample mounting and alignment of the optical fiber^315^. Figure 7g shows an ‘align-and-shine’ photolithography process that can transfer microstructures from the mask fiber to target fibers^286^. Interference lithography is also applied to produce periodic nanopillars array on fiber facets^285^ (see Fig. 7g1). This approach has good resolution with simple experimental setup, but it is not applicable for metasurface with aperiodic or arbitrarily designed antennas. Furthermore, photolithography on fiber sidewall is also explored (as Fig. 7g2)^316,317^.

#### Preform-based fiber drawing

Fiber drawing of specific preforms can produce various metamaterial fibers with designed cross-sectional patterns extending over long lengths^267–269,277^, showing a promising inroad to high volume production. As is illustrated in Fig. 7h, a macroscopic preform assembled by stacking indium wires is applied to hot furnace and thermally drawn to produce microscopic features in the thin fiber^246^. During the process, the feature dimensions from the original preform can be reduced by order of magnitude^268^. The preform can be fabricated by assembly, drilling and 3D printing^264,318,319^. Currently demonstrated metamaterial fibers made by single drawling are mainly applied for THz and mid-infrared frequencies due to fabrication resolution^246,247,282^. Figure 7i shows metamaterial fiber preform with tin/soda-lime wire arrays and an exemplary transition region after drawling^282^.

To further produce structured fibers with subwavelength features, iterative drawling can be applied^320,321^ (see Figs. 7j, k1). After successive drawling, the diameter of the intra-fiber nanowires can be reduced from hundreds of microns to about 15 nm with good radial and axial uniformity^320^. As shown in Fig. 7k2, the drawn fibers can be further assembled into different metamaterial preforms with various spatial structure distributions for further thermal size reduction^321^. Despite these demonstrations are non-optical fibers, this technology may be further adapted to fabricate multifunctional optical metamaterial fibers by improving optical loss and structural quality.

## Plasmonic meta-devices for controlling surface waves

Distinctive from previous chapters discussing propagating modes in meta-structured dielectric waveguides and optical fibers, in this section we will elaborate on the excitation and manipulation of surface wave (SW) modes using plasmonic meta-structures.

As a comparison, the optical modes in conventional fibers and waveguides are electromagnetic (EM) guided modes based on total internal reflection. The size of these optical elements is thus still wavelength scale constrained by diffraction limit, which severely hinders the high demand on device miniaturization. In contrast, EM SWs, including surface plasmon polaritons (SPPs) and their low-frequency counterpart spoof SPPs, are also eigen EM modes but instead highly confined at the material interface, which can find numerous applications such as super-resolution imaging^209^, enhanced light-matter interactions^322^, high-integration optical circuits^323–325^, bio-^326^ and chemical-sensing^327^, thanks to their deep-subwavelength and local-field enhancement characteristics. While natural SPPs only exist in optical frequency, the so-called spoof SPP modes are created in terahertz and microwave frequencies via patterning the highly conducting metal surfaces with subwavelength structures^328,329^. Recently, various meta-devices, including meta-coupler, meta-waveguide and circuits, are proposed to control SWs, providing us versatile possibilities for future on-chip optoelectronic applications^150^. Next, we will introduce the recent advances in SPP excitation and wavefront manipulations using these meta-devices.

### Excitations of surface waves using plasmonic meta-waveguide couplers

To utilize the novel properties of SW modes, the first step is to excite them efficiently. Unfortunately, because of momentum mismatch, the impinging free-space light cannot be directly coupled to SWs in general. To this end, various conventional optical devices were proposed, including prism coupler and grating coupler^330,331^. However, these elements are either too bulky and/or low-efficiency, which greatly hampers their further applications in integrated photonics^332^.

**Fig. 8:**
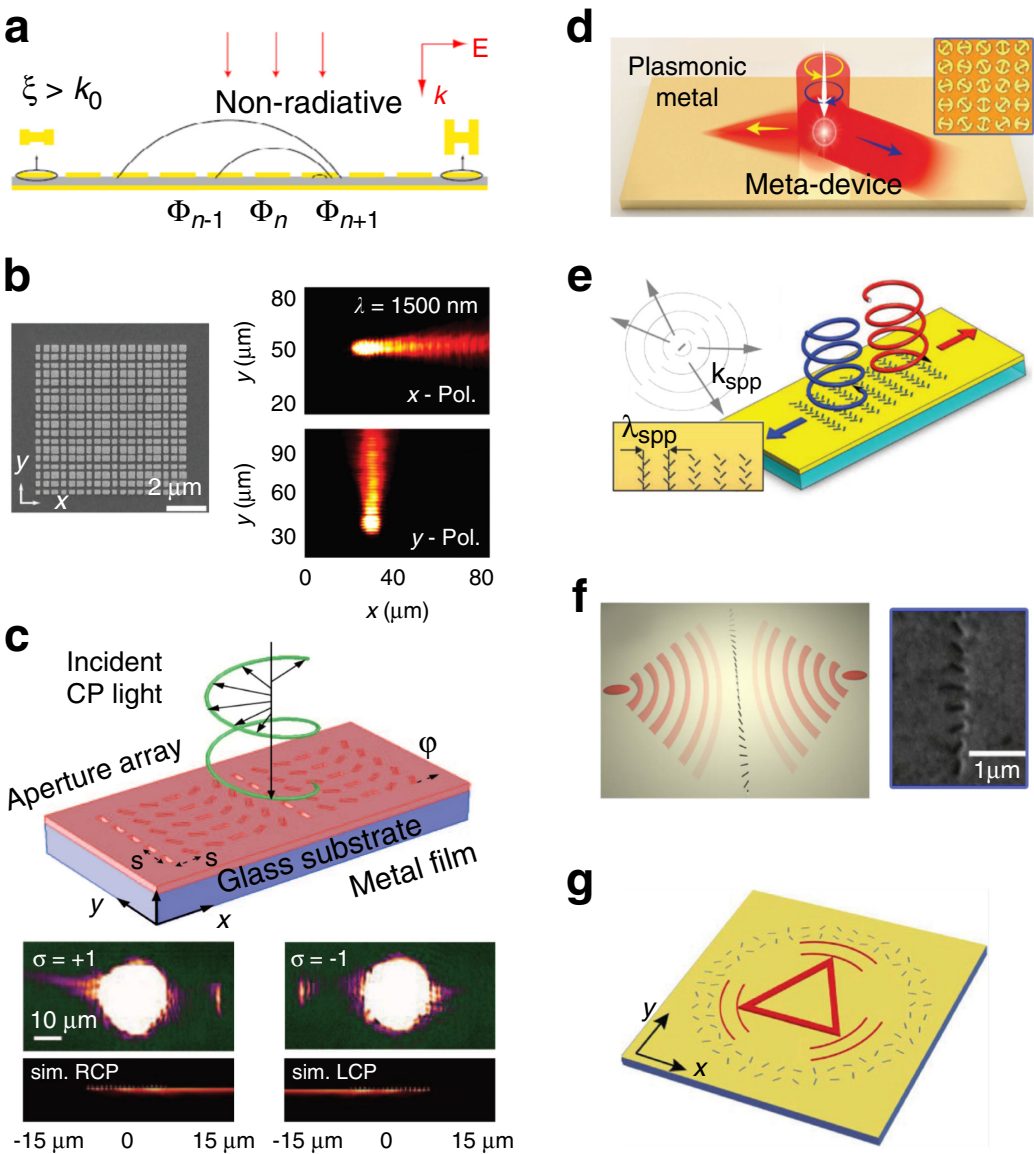
Excitations of surface waves using meta-structures. **a** Physical principle of PW-SW conversion by phase-gradient metasurface^70^. **b** Polarization-dependent directional SPP excitation at telecom wavelengths^115^ . **c** Helicity-dependent directional SPP excitations via Pancharatnam–Berry (PB) phase metasurfaces^113^. **d** Schematic of terahertz Metasurfaces for spin-delink spoof SPPs excitation combining the resonant and PB phase gradient. Inset: Partial picture of the fabricated meta-structure^119^. **e** Schematic of aperture-based SPP meta-coupler for polarization-modulated directional SPP coupling. Inset: image of multiple parallel column aperture pairs spaced *λ*_SPP_ apart^112^. **f** A plasmonic meta-slit that can achieve flexible SPP focal length switching by the helicity of input circular polarized (CP) light^122^. **g** Ring-shape array of nano-slits for SPP hologram^337^. Panels adapted from: Springer Nature (**a**^70^, **b**^115^, **c**^113^); Wiley (**d**^119^, **g**^337^); AAAS (**e**)^112^; OSA (**f**)^122^.

In 2012, Sun et al. proposed a new strategy to couple free-space propagating waves (PWs) to localized SWs based on a gradient metasurface in metal-insulator-metal (MIM) configuration^70^. As the meta-atoms are illuminated by EM waves, anti-parallel currents will be induced inside two metallic layers, creating a magnetic resonance. Via carefully tuning their local geometric parameters, such metasurface can provide a linearly changed reflection phase with its gradient denoted as $\xi = {{{\mathrm{d}}}}\varphi {{{\mathrm{/}}}}dx$. While the reflection phase changes slowly (i.e., *ξ* < *k*_0_, and here *k*_0_ denote the total wavevector of EM waves in vacuum), the metasurface will deflect the impinging wave to non-specular direction. Nevertheless, as *ξ* is large than *k*_0_, the metasurface cannot construct a far-field EM wave with an equal-phase plane, thus achieving PW-SW conversion, as shown in Fig. 8a. Both of the anomalous reflection and SW conversion effects can be described by the following equation:

6 $$k_x^r = k_0{{{\mathrm{sin}}}}\theta _i + \xi$$

where $k_x^r$ represents the parallel wavevector of reflection beam and *θ*_*i*_ is the incident angle of impinging waves. It implies that the gradient metasurface will introduce an additional wavevector to input EM waves, addressing the momentum mismatch issue between PWs and SWs. Considering that the generated SWs on such inhomogeneous metasurface are not eigen EM modes, the authors further constructed a SPP meta-coupler via connecting a mushroom structure with the metasurface that can guide out the “driven” SWs to eigen spoof SPPs. Such generic idea was soon realized based on other meta-structures working at various frequency regimes^115,116,333^. For instance, a polarization-dependent directional SPP meta-coupler working at telecom wavelengths was experimentally demonstrated^115^. The building block consists of a gold nanopatch and a thick gold film that are separated by a 50 nm-thick glass spacer. Via carefully designing both widths of atop nanopatches, they constructed two-dimensional gradient-phase metasurface that can couple input *x*- or *y*- polarized PWs to eigen SPPs propagating along two orthogonal directions, as depicted in Fig. 8b.

Apart from the resonance-based metasurfaces, Pancharatnam–Berry (PB) phase metasurfaces are also exploited to achieve helicity-dependent directional SPP excitations. As shown in Fig. 8c, a transmissive metasurface is composed by the elongated aperture array with constantly varied orientation angles^113^. Except for Bragg reciprocal wavevector, such metasurface can also provide an additional geometric phase based wavevector for impinging light, achieving the helicity-dependent directional SPP excitations at near-infrared wavelengths. While the handedness of input CP light is flipped, the excitation direction of SPP will be switched to opposite direction. In a following work, a MIM typed PB meta-coupler is further demonstrated to couple SPPs with their amplitude and phase independently modulated by the polarization state and polarization angle of incoming light^114^. However, the early PB meta-couplers usually suffer from low-efficiency issue. To address such challenge, a new scheme is reported to solve two fundamental issues widely existing in previous studies^334^. First, via carefully optimizing the polarization conversion efficiency of the building block, the PB meta-coupler is able to convert impinging EM waves to driven SWs with nearly 100% efficiency. Second, the guided-out plasmonic metal should be carefully designed to support both TM and TE spoof SPP mode, solving the polarization mismatch issue between input CP wave and guided-out SPPs. A realistic meta-coupler working in the microwave regime is designed and fabricated, exhibiting a high spoof SPP excitation efficiency of over 80%.

While PB phase metasurfaces possess the advantages such as dispersionless phase response and easy design/fabrication, they still encounter a strong restriction of spin-correlated functionalities. To overcome this limitation, Mueller et al. proposed an approach to design a single metasurface carrying two arbitrary and independent phase distributions for the input lights of two orthogonal polarization states (including linear, circular and elliptical ones). The combination of propagation phase and PB geometric phase enables us to delink two functionalities of the metadevices for two orthogonal polarizations^152^. Various multifunctional meta-structures leveraging Jones matrix toolbox are demonstrated for achieving either SPP excitations or non-specular beam deflections modulated by the spin states of incoming CP lights^118,335^. A new design scheme was soon proposed to simultaneously achieve high efficiency surface plasmon excitations and wavefront controls based on a single meta-coupler^119,120^. Except for designing a linear-phase profile along *x* direction, which is mainly responsible for surface plasmon excitations, they also added a specific phase profile along *y* direction to simultaneously modulate the wavefronts of generated SPPs. Via combining both resonance phase and PB phase together, such meta-coupler can achieve either SPP focusing, or deflection effect shined by different spin-polarized EM waves (see Fig. 8d).

Except for the meta-couplers based on wavevector compensation scheme, narrow apertures are also widely utilized to launch SPP modes depending on diffraction effect^336^. In 2013, an aperture-based SPP meta-coupler was proposed^112^ to realize polarization-modulated directional SPP coupling. For a single aperture, it approximately behaves like an in-plane dipole source that can emit SPP waves along both sides but with a phase difference of π. As illustrated in Fig. 8e, such meta-coupler can enable constructive or destructive interference of SPP on two opposite sides via carefully designing the individual orientation angles and spatial separation of the aperture pairs. Such aperture-based metasurfaces can not only launch directional SPPs, but also tailor their wavefront simultaneously^122,337,338^. For instance, Fig. 8f depicts a plasmonic meta-slit that can achieve SPP focusing with its direction and focal length flexibly switched by the helicity of input CP light^122^. The meta-devices are composed of single or double arrays of subwavelength nano-slits with their orientation angles satisfying the parabolic distribution. To construct more complicated near-field patterns, the double-lined metasurface was adopted to modulate both the local amplitude and phase of generated SPP via controlling the tilted angles of nanoslit pair. Benefiting from these two degrees of freedoms, the plasmonic SPP caustic curves, Airy beam, and complex holography (Fig. 8g) are successfully demonstrated^337^.

### Manipulations of surface waves using plasmonic meta-waveguides

In the previous section, we have discussed how to employ meta-couplers to efficiently convert free-space light to near-field SPPs. For the future integration photonic applications, versatile on-chip manipulation the SPP beams is highly desirable, including SPP guidance, wavefront tailoring, and far-field emission.

#### Surface wave waveguides and circuits

**Fig. 9:**
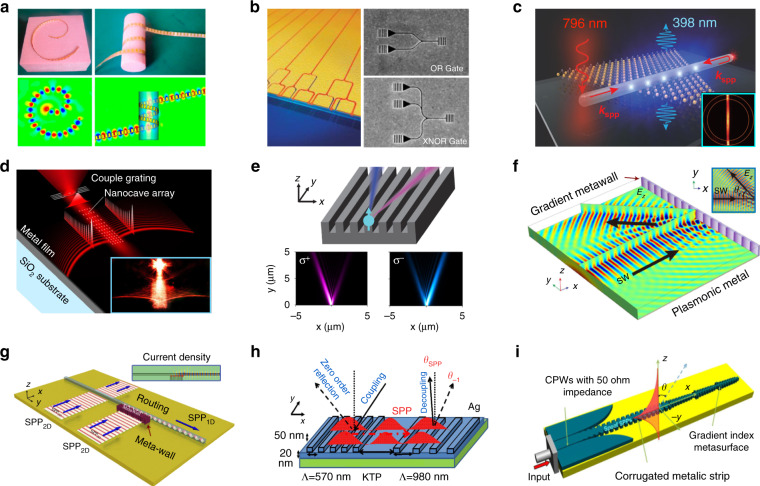
Manipulations of surface waves via meta-structures. **a** Spoof SPP transmission lines constructed by subwavelength comb-shaped corrugated metal strips^344^: Fabricated samples (first row) and simulated electric field distributions (second row). **b** All-optical nanoscale SPP logic gates: Schematics (left panel)^347^ and representative SEM images (right panels). **c** SPP silver nanowire waveguide integrated with a single layer of MoS_2_ for second harmonic generation. Inset: Fourier imaging microscopy collecting signals^348^. **d** Inhomogeneous nanohole array fabricated on the silver film for SPP Airy beam generation by non-perfectly matched diffraction^354^. **e** Hyperbolic metasurface for SPP PSHE in visible regime^124^ . **f** Gradient-phase meta-wall for spoof SPP anomalous reflection. Inset: simulated abnormal spoof SPP reflection results in *x*-*y* plane^73^. **g** Schematic of the transmissive metawall router for the transformation of 2D SPP to 1D SPP in waveguides. Inset: Simulated current density in plasmonic router^71^. **h** SPP hologram meta-grating for vortex beam generation^362^. **i** SPP decoupling via phase-gradient metasurface^127^. Panels adapted from: PNAS (**a**)^344^; ACS (**b**^347^, **c**^348^); APS (**d**^354^, **f**^73^, **h**^362^); Springer Nature (**e**)^124^; De Gruyter (**g**)^71^; AIP Publishing (**i**)^127^.

Massive plasmonic meta-structures can be applied to construct meta-waveguides for SW transports and controls in deep- subwavelength scale, such as V-shaped grooves, hybrid nanowires, and plasmonic tapers^339–341^. In low-frequency counterpart, spoof SPP waveguides were also developed based on various structured surfaces^328,342,343^. In particular, an ultrathin and flexible comb-shaped plasmonic surfaces is reported to transport the conformal surface plasmons (CSPs) at the microwave regime^344^. As shown in Fig. 9a, such CSP modes can be bent, folded and even twisted with very low propagation and absorption losses on such surfaces with various curved configurations.

Considerable efforts have also been devoted to SPP based integrated optical circuits. In 2011, a simple double-nanowire network was proposed to perform compact logic operations, via illuminating the two nanowire terminals by excitation lasers of specific polarizations and relative phases. Based on the plasmonic interference effect, such plasmonic network can direct guided SPP waves to corresponding output, exhibiting various functionalities of beam splitting, routing and switching^345^. Soon, a binary NOR gate was experimentally verified in a plasmonic network of four-terminal nanowires via cascading OR and NOT gates^346^. Meanwhile, several high-integration all-optical logic gates were developed (including OR, NOT, XOR, and XNOR ones) based on plasmonic nanoslot waveguides, exhibiting high intensity contrast and lateral confinement (see Fig. 9b)^347^.

The local-field enhancement effect in plasmonic devices sheds light on many on-chip photonic applications, such as tunable sources, nonlinear microscopy, all-optical switching, etc.^348–351^. For instance, Li et al.^348^ experimentally demonstrated the second harmonic generation (SHG) in a plasmonic nanowire waveguide (Fig. 9c). The sample consists of a crystallized silver nanowire and a single layer of MoS_2_ with large second order susceptibility. Shined by the excitation laser, the excited SPP mode will propagate and then be reflected back inside the nanowire. For these two counter-propagating SPPs, the momentum matching condition is automatically addressed, thus facilitating the nonlinear effect inside such system.

#### Wavefront control of surface waves

While some basic optical elements (e.g., dielectric triangular prism and cylindric lens) are initially adopted to control the propagation of SPP, they usually possess bulky size and limited functionalities^352^. Relying on Bragg scattering effect, the concept of plasmonic crystals was proposed to realize SPP beam reflection, splitting, interference and demultiplexing^347,353^. To construct more complicated near-field wavefronts, the non-perfectly matched diffraction effect is employed to generate SPP Airy beam and experimentally demonstrate its non-diffracting and self-healing properties, as shown in Fig. 9d^354^. An inhomogeneous nanohole array is fabricated on the silver film to provide a nonlinear phase modulation for input SPP. In addition, various proposals such as transformation based devices^355,356^ and holographic plates^357,358^, also showcase diverse opportunities for on-chip SPP manipulations.

The rapid development of plasmonic meta-waveguides has also opened up a new direction for near-field manipulations. In 2013, Liu and Zhang proposed theoretically a plasmonic metasurface constituted by metallic subwavelength grating that can support SPP modes with non-trivial (including flat and hyperbolic) dispersion relations. Several alluring phenomena such as anomalous-diffraction, non-diffraction and negative refraction of SPPs were numerically demonstrated^123^. As illustrated in Fig. 9e, such concept of hyperbolic metasurfaces for surface plasmon controls was soon experimentally verified in visible regime^124^. Other than these bulky meta-deivces, Dong et al. proposed a new concept to design ultrathin gradient metawalls (i.e., near-field version of metasurfaces) to achieve the arbitrary wavefront tailoring of SPP, such as SPP anomalous reflection (Fig. 9f), near-field focusing and Bessel beam generations^73,359^. Such meta-wall is constituted by ultrathin gradient-index microstructures and a metallic plate that can totally reflect incoming SPPs with specific reflection phases profile, leading to the versatile wavefront reshaping of SPP. This idea was soon further developed to achieve one-dimensional SPP line wave excitations with a transmissive metawall (see Fig. 9g)^71^. This find may be applied in modulating on-chip photonic networks.

#### Out-of-plane emission of surface waves

For numerous photonic applications, we also need to decouple near-field SWs to far-field PWs in a desired manner. While encountering some discontinuous interface (e.g., bump, hole, groove), SW can be partially scattered to free space but with the radiation intensity and direction hardly controlled^360^. Periodic apertures are utilized for achieving directional out-of-plane emission of SPP^361^. As shown in Fig. 9h, holographic images can be created by incorporating the computer-generated hologram technology with bragg meta-grating to decouple SPP to free-space^362^. Other complex wavefronts, like accelerating Airy beam, focused beam, vortex beam are also suitable for this method^363–365^. In addition, Fig. 9i shows H-shaped gradient-phase metasurfaces to guide the spoof SPP modes on metallic strips to directional radiative waves^127^. According to the generalized Snell’s law, the emission direction can be flexibly modulated by the phase gradient of the metasurface. Further utilizing the dispersive nature of spoof SPP mode, a broadband frequency scanning antenna is created based on such gradient metasurface, exhibiting a continuous scanning range from 4.8° to 37.2° operating at 8.8–10.7 GHz^128^.

## Inverse-designed metamaterial waveguides

Forward-designed metamaterial discussed in previous sections generally starts from a well-established library of structure templates, followed by customized tuning with a handful of specific parameters based on applications. Despite the clear physical guidelines offered by these intuition-based strategies, the device structures are usually not fully optimal and do not consider the massive design space of irregular structures. Furthermore, some sophisticated functionalities such as ultracompact and multifunctional devices are hard to design using forward design methods^15^.

To settle these challenges, various numerical algorithms have been developed that allow a computer to design and optimize freeform metamaterial waveguide structures with dramatically boosted degrees of design freedom to maximize device performance. These computer algorithm-based inverse-design strategies offer a potentially effective and automated method to ‘design-by-specification’^15,147^, in a manner following computer aided design techniques that are well established in the integrated circuit design community.

**Fig. 10:**
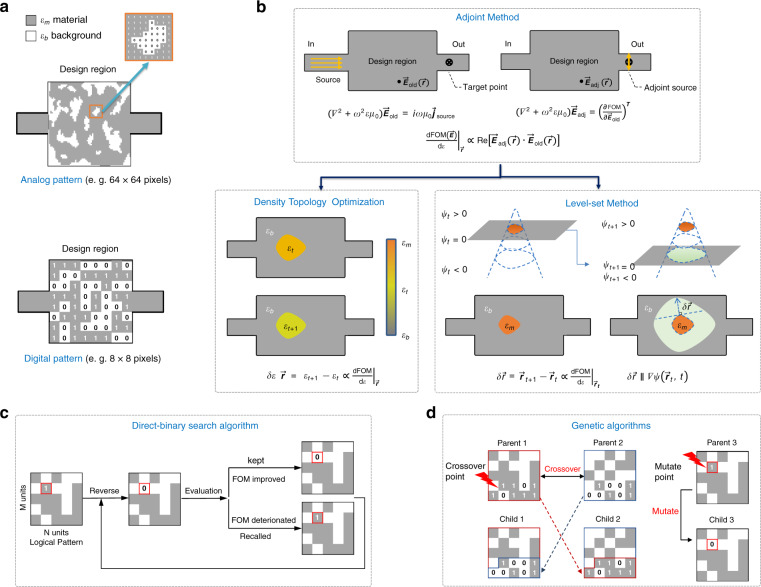
Inverse-design tutorials for meta-waveguides. **a** Binary etching states for analog and digital meta-waveguide patterns. Pixel dimensions of an analog pattern are much smaller to produce curvilinear device boundaries. Digital patterns have relatively big regular-shaped (e.g. rectangular or circular) pixels^62^. **b** Adjoint method schematic: two simulations are needed for each iteration^15^, a forward simulation using the incident source as excitation and an adjoint simulation using reciprocal displacement current $\frac{1}{{{{{\mathrm{i}}}}\omega \mu _0}}\left( {\frac{{\partial {{{\mathrm{FOM}}}}}}{{\partial {{{\mathbf{E}}}}_{{{{\mathrm{old}}}}}}}} \right)^T$ as excitation source, where vector **E**_old_ is the value of the electric field at a given point before update. Sources for each simulation are marked in yellow. For level-set method, each point vector **r** on the boundary moves along its normal direction with a speed equivalent to the gradient value at this point. **c** Illustration of the iterative procedure in DBS algorithm. **d** The crossover and mutate mechanisms in a GA for reproducing new population.

Inverse-designed metamaterial waveguides can be classified into two categories: analog and digital^62,67–69^. To design a device, the design region is first specified and then discretized into *M* × *N* unit shapes, called “pixels”. The “pattern” is thus considered as a matrix of the pixels with permittivity distribution of [*ε*]_*M*×*N*_, where each pixel (*i*, *j*) has a permittivity of *ε*_*i,j*_ accordingly. Then, algorithms are employed to find an optimized permittivity distribution to fulfill figure-of-merit (FOM). As exemplified in the upper panel in Fig. 10a, analog metamaterial waveguides generally have relatively small pixels^366–368^ $\left( { \ll\!\! \lambda {{{\mathrm{/}}}}10} \right)$ to meet the perturbation theory^369^. Thus, the permittivity *ε*_*i,j*_ or its shape information can vary continuously to calculate the gradients information of the FOM (discretization is conducted later to produce binary devices)^15^. In contrast, pixel dimensions of a digital metamaterial waveguide are much larger (~*λ*/10) (lower panel in Fig. 10a)^68,370^, and the permittivity *ε*_*i,j*_ is restricted to binary distribution, e.g. corresponding to the logical “1” or “0” state.

In this section, we present explicit design tutorials and highlight representative applications using inverse-deigned metamaterial waveguide platforms. Diverse design algorithms are outlined and compared in terms of its versatility and applicability, including gradient-based, non-gradient-based and deep learning-inspired optimization methods.

### Analog metamaterial waveguides

**Fig. 11:**
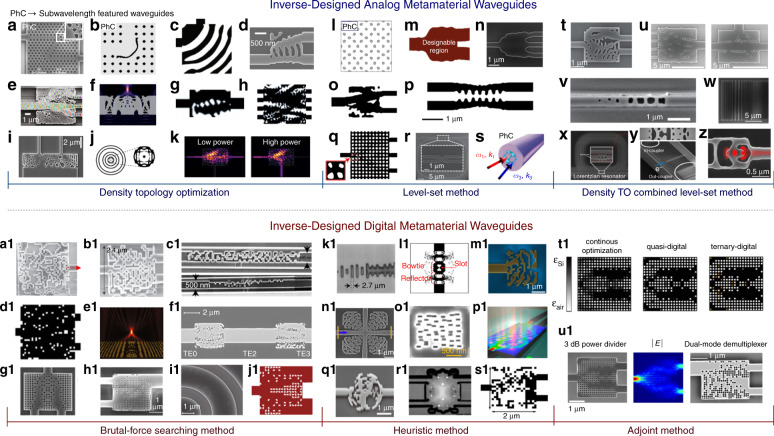
Inverse-designed analog (**a**–**z**) and digital (**a**1–**u**1) metamaterial waveguides. **a**–**k** Analog meta-waveguides inversely designed by density topology (TO) optimization. Note: non-subwavelength structures, like photonic crystals (PhC), are marked out. **a** A PhC waveguide Z-bend^373^ and **b** 90° bend^374^. **c** Metamaterial waveguide 90° bend^375^. **d** Polarization beam-splitter (PBS)^376^. **e** 3-channel mode multiplexer (MUX)^377^. **f** Planar nanolens^378^. **g** **TE**_**0**_**-TE**_**1**_ mode converter. **h** 3 × 3 hub^58^. **i** 2-channel wavelength de-MUX^387^. **j** Nonlinear microcavity^388^, **k** Optical Kerr switch^366^. **l**–**s** Level-set method. **l** A PhC with maximizing band gaps^390^. **m** 3 dB^367^ and **n** 1 × 3 power splitter. **o** Dual-channel mode MUX. **p** 2 × 2 power splitter. **q** 3-channel wavelength de-MUX^391^. **r** Cavity-waveguide coupler^392^. **s** Nonlinear PhC fiber^393^. **t**–**z** Hybrid method. **t** Wavelength de-MUX^368^. **u** Mode MUX^395^. **v** Fabry-Perot microresonator^396^. **w** Broadband chip-fiber grating coupler^394^. **x** Non-reciprocal pulse router^397^. **y** Laser-driven particle accelerator^398,540^. **z** Vertical coupler^399^. **a**1–**j**1 Digital meta-waveguides designed by direct-binary search (DBS) algorithm. **a**1 Waveguide coupler^67^. **b**1 PBS^68^. **c**1 Waveguide cloak^64^. **d**1 1 × 2 power splitter with arbitrary splitting ratio^409^. **e**1 Planar nanolens for focusing Bloch surface wave^410^. **f**1 Cascade multimode converter^411^. **g**1 3 dB power splitter^370^. **h**1 Dual-channel mode MUX^415^. **i**1 3-channel mode bend^69^. **j**1 1 × 2 mode converter^422^. **k**1–**q**1 Heuristic methods. **k**1 Fiber-to-waveguide coupler^423^. **l**1 Planar resonator^424^. **m**1 Broadband reflector^425^. **n**1 Wavelength pass filter^426^. **o**1 Wavelength router^427^. **p**1 Programmable light modulator^428^. **q**1 OAM emitter^47^. **r**1 Power splitter^429^. **s**1 PBS^430^. **t**1 Adjoint method procedures for digital meta-waveguides^62^. **u**1 Design examples^62^. Panels adapted from: AIP Publishing^374^; IEEE^375,394,395^; OSA^58,62,67,367,370,373,377,388,392,393,396,409,415,422–429,540^; De Gruyter^387^; SPIE^376^; ACS^366,378,411^; Springer^390^; Springer Nature^47,64,68,69,368,391,397,399,410^; Elsevier^430^.

Analog metamaterial waveguides using sufficiently small pixels may have tremendous degrees of design freedom. To manage and explore this high dimensional design space in a computationally tractable manner, two classes of gradient-based optimization methods are typically considered: density topology optimization (TO) and level-set method, as shown in upper panels of Fig. 11. These methods, based on the adjoint method, are computationally scalable to systems comprising thousands and even millions of pixels because the number of simulations required to calculate gradients is decoupled from the total number of pixels^147,367^, which vastly broadens the generality and computational efficiency of inverse design. Gradient-based adjoint methods were introduced into nanophotonics in the early 2000’s by researchers from the mechanical engineering^371^ and control theory^372^ communities, where the adjoint method was established.

#### Density topology optimization

##### Design procedures

(i) The permittivity values *ε*_*i,j*_ of each pixel are randomized and relaxed to span a continuum of values. (ii) Then, gradient descent algorithms perturbatively adjust the permittivity values at each pixel in a manner that improves the FOM, producing an analog pattern (shown in the bottom left panel of Fig. 10b). (iii) The continuous permittivity values of each pixel obtained in Step (ii) are pushed towards binary values, where only two boundary values can be chosen, and the FOM is reevaluated. Iterations are thus formed by going back to Step (ii) and (iii) to produce a new binary pattern.

##### Foundational results

Initial studies in the density topology optimization of nanophotonic devices are most linear devices, where the electromagnetic field satisfies the Hermitian condition. These demonstrations include, e.g. photonic crystal (PhC) waveguide Z-bend^373^ (Fig. 11a) and 90°-bend^374^ (Fig. 11b), where the optimized structure shows a significantly lower loss for a wider range of frequencies compared to forward designs. Density topology optimization has since been exploited to inversely design various waveguide devices with free-formed subwavelength features (Fig. 11c–f), including, for instance, high-performance 90° waveguide bend^375^, polarization beam splitter^376^, three-channel mode multiplexer^377^, and nanolens with structural integrity^378^. More recently, density topology optimization was used to tailor guided-mode phenomena in freeform metasurfaces, leading to high efficiency^379–381^ and multifunctional devices^382,383^. New diffraction phenomena are enabled as well by analyzing these complex mode scattering dynamics^384^.

In contrast to typical TO schemes, an “objective first method” was proposed to allow both electromagnetic filed and permittivity values to optimize individually^385^, which allows for non-zero physics residuals. Alternating directions method of multipliers (ADMM) algorithm were employed in the optimization process without using the adjoint method. A wider range of functional devices were investigated using the method^58,386,387^, including mode converter (Fig. 11g), hub (Fig. 11h), fiber coupler, optical diode, and polarization beam splitter, as well as multi-function device handling simultaneous focusing and wavelength demultiplexing (Fig. 11i).

For nonlinear devices, the electromagnetic field no longer satisfies the Hermitian condition and adjustments to the adjoint method are required. A 2D microcavity was designed for nonlinear frequency conversion by solving a linear adjoint equation with time-dependent material^388^ (Fig. 11j). Simplified nonlinear optimization was then explored based on a linear adjoint equation with time-invariant material^366^, followed by devising a compact photonic switch in Kerr nonlinear material (Fig. 11k).

#### Level-set method

##### Design procedures

(i) The boundary of the material subjected to *φ*(**r**) = 0 is allowed to vary continuously. During the optimization process, the boundary is time dependent and can be expressed as the zero solution of the level-set function .

7 $$\psi \left( {{{{\mathbf{r}}}},t} \right) = \varphi \left( {{{\mathbf{r}}}} \right) + h\left( t \right)$$

The negative solution and positive solution of *ψ*(**r**, *t*) correspond to the regions filled with permittivity of two boundary values respectively. (ii) Then, the evolution of the boundary can be considered as a change of the level-set function with respect to *t* via gradients, as shown in the bottom right panel in Fig. 10b.

##### Foundational developments

A shape derivative method was proposed based upon the adjoint variable method and perturbation theory in the early 2000’s^389^, which enables the gradient descent algorithm based on shape derivative method. Level-set method is mathematically distinct from the shape derivative method, but essentially equivalent. Initial studies in the level-set method involved bandgap engineering of PhC devices^390^, as shown in Fig. 11l. Later research validated the feasibility of using this method to devise metamaterial waveguides. By parameterizing the boundary of the material with the level-set method, a high-performance silicon Y-junction splitter was designed in an ultra-compact area^367^ (Fig. 11m). A general level-set method was proposed for subwavelength featured waveguides that directly incorporates fabrication constraints, especially for curvature constraints on device boundaries^391^. A 1 × 3 splitter, mode demultiplexer, directional coupler, wavelength demultiplexer, and cavity-waveguide coupler^391,392^ were later reported (Fig. 11n–r). Besides, level-set method was used to design nonlinear waveguides, such as PhC fibers with large nonlinear frequency-conversion efficiencies (Fig. 11s)^393^.

#### Density topology optimization combined level-set method

##### Design procedures

(i) First, an optimized analog structure with gray permittivity is obtained using TO method. (ii) Then, the structure is binarized employed level-set method. The level-set method could theoretically prevent intermediate gray structures.

##### Foundational developments

Figure 11t shows a wavelength demultiplexer with wide bandwidths designed by simultaneous TO and level-set method^368^. The method was also used to design general 1D grating couplers without any human input from start to finish (including a choice of initial condition)^394^, as depicted in Fig. 11w. Various functional integrated devices had also been demonstrated as Fig. 11u–z^395–399^, such as mode multiplexer and mode splitter, Fabry-Perot micro-resonator, non-reciprocal pulse router for chip-based LiDAR, laser-driven waveguide-integrated particle accelerator, and diamond photonic waveguides.

#### Fabrication constraints for gradient-based algorithms

Despite the benefits discussed above, such analog devices nevertheless encounter challenges in terms of fabrication constraints. The TO method results in intermediate “gray” structures, but only binary permittivity distribution can be efficiently implemented in experiments for most cases. A wide variety of proposals are reported to mitigate this issue of “gray” areas in structure, such as density filters^400^, sensitivity filters^401^, penalty functions^402^, artificial damping^403^, and temperature parameter trick^404^.

In level-set method, small features, such as narrow gaps or bridges, are usually inevitable. A typical approach is to exploit shape parameterizations that automatically satisfy the desired minimum feature constraint, known as a geometry projection method^405^. Another approach is to evaluate a dilated and eroded version of the device during the optimization process with improved fabrication robustness and length scale constraint^406^. Curvature limit techniques are also useful for the removal of narrow gaps^391^. To enforce hard design constraints, methods based on reparameterization have been developed in which geometric parameters are initially defined in a continuous latent space and then mapped onto a physical device^407^. This mathematical mapping is specified such that devices that violate a given physical constraint, such as minimum feature size, are not even considered. This method has the advantage not only of enforcing hard constraints, but also of simplifying the design space and improving the reliability of the optimization process through elimination of non-compliant device patterns.

### Digital metamaterial waveguides

Analog metamaterial waveguides utilize curvilinear device structures with high degrees of freedom but entail high fabrication resolution requirements. In contrast, digital patterns that utilize regular arrays of spatially coarse pixels can be designed and fabricated in an easier fashion. To date, most popular optimization methods for designing digital metamaterial waveguides fall into three classes: brute-force searching, heuristic, and adjoint methods, as shown in lower panels of Fig. 11.

#### Brute-force searching method

##### Design procedures

Take the direct-binary search (DBS) algorithm as an instance. (i) Fist is reversing the logical state of a randomly chosen or specifically designed pixel and evaluating the FOM. (ii) If FOM is improved, the new pixel state is kept and the next pixel is reversed; otherwise, the original state is recalled (Fig. 10c). (iii) One iteration ends when all the pixel states are inspected, and the final pattern is set as the initial one of the next iteration.

##### Foundational developments

DBS algorithm is a representative brute-force searching method introduced from digital hologram communities^408^. Massive digital metamaterial waveguides were invigorated using DBS algorithm, such as free-space- to-waveguide coupler^67^ (Fig. 11a1), polarization beamsplitter^68^ (Fig. 11b1), waveguide cloak^64^ (Fig. 11c1), power divider^409^ (Fig. 11d1), nanolens^410^ (Fig. 11e1), and mode converter^411^ (Fig. 11f1), optical diode^66^, 180° waveguide bend^65^, polarization rotator^63^, active optical switch^412^, polarization splitter-rotator^413^.

In Fig. 11g1, a photonic-crystal (PhC)-like metamaterial structure was proposed for 3 dB power divider that exploits partial-filling hole pixels to suppress the etch depth fluctuations caused by the reactive ion etching (RIE) lag^414^ and thus helps to improve fabrication tolerances^370^. A 5 dB performance increment was achieved compared with a device of similar size optimized based on full-filling rectangle pixels. Various PhC-like waveguides were also reported, including mode multiplexer^415^ (Fig. 11h1), on-chip routing^69^ (Fig. 11i1), star crossing^416^, wavelength demultiplexer^417^, polarization rotator^418^, multimode bend^419^, dual-mode crossing^420^, and power splitter^421^. Furthermore, a rotatable DBS algorithm that adds a calculation of rotational dimension was proposed as Fig. 11j1 for simultaneous mode converting and power splitting^422^.

#### Heuristic method

##### Design procedures

Take the genetic algorithm (GA) as an instance. (i) Generating initial populations as the first generation. Each population is a matrix of pixel distribution. (ii) Evaluating each population’s FOM. (iii) Producing new populations with sequential application of selection, crossover, and mutation. Multiple strategies can be selected, e.g., roulette wheel or tournament selection. Each of the selected population has a specified or random probability to have a crossover with another population, where the crossover point is randomly picked (the left panel in Fig. 10d). After crossover, each population in the new generation has a probability of mutation, where a random number of pixels are selected to reverse (right panel of Fig. 10d). Iterations are thus formed by going back to Step (ii) to evaluate the new generation and population reproduction in Step (iii).

##### Foundational developments

As DBS algorithm mostly converges to a local optimum, it is more suitable for optimizations in a small parameter space. With a large parameter space, heuristic method, such as genetic algorithm (GA) and particle swarming optimization (PSO), are more competitive. Fig. 11k1 sketched a GA-optimized SiO_2_/SiON telecom-fiber to ridge-waveguide coupler^423^, with a 2 dB enhancement in efficiency compared with direct coupling. As shown in Fig. 11l1–p1), GA was then extended to a wider range of technologically relevant applications, such as silicon planar resonator^424^, reflector^425^, long-pass filter^426^, wavelength router^427^, and light modulator^428^. More recently, GA method was combined with simulated-annealing algorithm to enable accurate phase control in freeform meta-waveguides, contributing to high efficiency devices for complicated mode conversion from guided modes to free space (e.g., orbital angular momentum emitter^47^ shown in Fig. 11q1).

On the other hand, the particle-swarm optimization algorithm also can be applied to design metamaterial waveguides (see Fig. 11r1, s1), such as 2 × 2 power splitter^429^ and polarization beam splitter^430^. Particle-swarm optimizations are based on the movements of a population of candidate solutions (particles) in the search space. During optimization, the initially distributed particles continue moving towards the then-current optimum particle in the swarm, until the FOM is reached^431^.

#### Adjoint method

##### Design procedures

(i) Tuning the permittivities of all pixels with a fixed shape continuously and individually to obtain an optimized analog pattern with “gray” pixels using adjoint method. (ii) Then, a forced biasing approach was applied to convert the analog pattern to a “quasi-digital” one in which the permittivities of most pixels are close to two boundary values. (iii) Fabrication-constraint brute-force quantization methods were designed to transform the “quasi-digital” pattern into an “*N*-ary digital” pattern, as illustrated in Fig. 11t1.

##### Foundational developments

Distinctive from the TO or lever-set method, gradient information is generally hard to retrieve for digital meta-waveguides. Without gradients to efficiently update searching direction, convergence of these optimization algorithms is often considerably slower. Fortunately, recent research validated the feasibility of adapting the adjoint method into the inverse design of digital meta-waveguides. As shown in Fig. 11u1, a 3 dB power divider and a dual-mode multiplexer were demonstrated^62^, using PhC-like subwavelength structure with cylindrical-shaped base pixels in design region. At the implementation stage of “*N*-ary digital” pattern, cylinders with different “gray” permittivities in the “quasi-digital” pattern are replaced with air cylinders with original radius, silicon cylinders, or air cylinders with *N* − 2 different radii on the basis of effective medium theory to minimize the performance degradation due to the digitalization process. Compared with the conventional DBS method, the introduction of adjoint method can improve design efficiency by about five times, while the performance optimization can reach approximately the same level.

### Deep learning enabled inverse design in metamaterial waveguides

Artificial neural networks (ANN) are algorithms that utilize a series of nonlinear mathematical functions, in the form of neurons, to specify highly nonlinear mappings between inputs and outputs^148^. A training process, in which weights within the neurons are iteratively adjusted, is used to tailor these mappings. ANNs have been a topic of study for the last half century and have gained immense popularity in nearly every technical field of research over the last decade due to the emergence of the deep network framework, user-friendly software packages, and application-specific hardware^432,433^. The deep ANN framework, where many layers of neurons are utilized in the algorithm, has led to particularly versatile and expressive algorithms that can serve as universal functional approximators. Software packages, ranging from PyTorch to TensorFlow, have led to standardized and easy-to-program interfaces to implementing ANNs with arbitrary architectures^432^. Specialized hardware includes graphical and tensor programming units that can dramatically speed up the network training process. Furthermore, the culture of opening and sharing in the machine-learning community leads to the availability of many state-of-the-art algorithm resources easy to access and follow up online^432,434^ (https://github.com/iguanaus/ScatterNet, http://github.com/yuruiqu/Transfer-learning_Data, https://github.com/PRGatech/DimensionalityReduction).

**Fig. 12:**
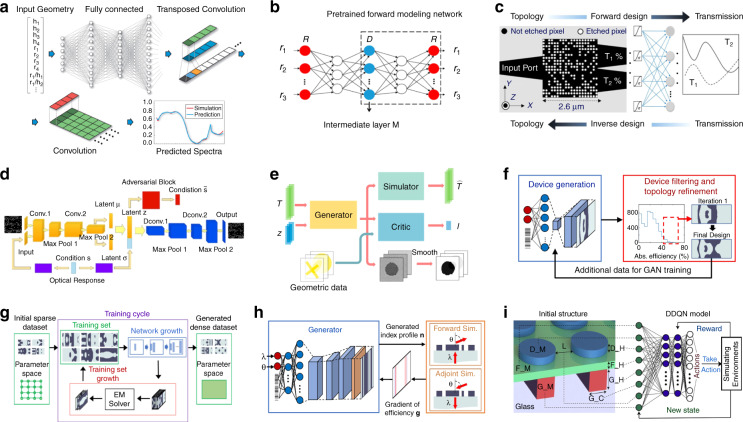
Artificial neural networks for nanophotonic inverse design. **a**–**c** Inverse design with discriminative ANNs. **a** A convolutional neural network (in conjunction with DBS algorithm) for designing all-dielectric metasurface^439^. **b** Tandem network (an inverse network connected to a pretrained forward modeling network) for designing multiple thin film stacks^440^. **c** Meta-waveguide inversely designed by residual deep neural network (ResNet)^487^. **d**–**h** Generative ANNs. **d** A conditional VAE network for designing analog power splitters with various splitting ratios^61^, where etch hole positions are the design space. **e** A GAN network for metasurface that supports tailored transmission spectral responses^448^, with three networks: the generator, the simulator, and the critic. **f** A conditional GAN network for designing meta-grating^449^. **g** A modified GAN network consisting of three principal features: progressive network growth, the self-attention mechanism, and progressive growth of the training set^450^. **h** A global topology optimization network (GLOnet)^452^. Optimization is done by coupling a generative network with direct electromagnetic solver. **i** Inverse design of metasurface hologram with reinforcement learning^457^. Initial structure used as the input double deep Q-learning network (DDQN) at each step. Panels adapted from: OSA (**a**)^439^; ACS (**b**^440^, **e**^448^, **f**^449^, **g**^450^, **h**^452^); Springer Nature (**c**^487^, **i**^457^); Wiley (**d**)^61^.

In this section, we give an overview of inverse-design methods based on discriminative networks, generative networks, and reinforcement learning. While we will present examples from the broader context of nanophotonics design (see Fig. 12), the concepts can readily apply to meta-waveguide design without loss of generality^148,432,435^.

#### Inverse design with discriminative ANNs

Discriminative ANNs are algorithms that can capture data relationships of the form *y* = *f*(*x*) When *x* specifies a device pattern and *y* is its optical response, the discriminative ANN serves as a surrogate electromagnetic (EM) solver, solving the forward problem^432^. To train a discriminative ANN, a training set of known device patterns and optical responses is first created using a conventional EM solver. The ANN is then trained to minimize error between the outputted and known optical responses, for given device pattern inputs. Mathematically, the loss function is defined to be the mean square error between the outputted and ground truth optical responses, and this function is minimized during the training process by applying back-propagation algorithm^148^. While the training data collection and ANN training are computationally expensive, a trained ANN can perform computations orders of magnitude faster than a conventional solver. A challenge to training an accurate and generalized ANN is curating a sufficiently large and diverse training set that properly represents the desired computation space. A basic and typical strategy is to use a random, uniformly distributed set of devices^432^. If there are known, statistically rare features pertaining to the device geometry or optical response, these features can be learned by the ANN by augmenting the training set with a disproportionally large number of examples of these data.

Discriminative ANNs can be used for inverse design in multiple ways. One is that the surrogate solver can be used in conjunction with conventional optimization methods, which include evolutionary strategies^436^, particle-swarm optimization^437^, and brute-force sweeping and searching^438,439^. Many of these strategies typically require hundreds to thousands of simulations performed in series, and the utilization of an accurate high-speed solver can reduce total optimization times from hours or days to seconds, one example is illustrated in Fig. 12a^439^. Another way is to use the trained forward ANN to facilitate the training of an inverse network, which attempts to output a device pattern given a desired optical response input^440^ (shown in Fig. 12b). The reason why the tandem network is trained, as oppose to direct training of an inverse network, is that the inverse problem is a one-to-many mapping problem: there exist multiple device patterns that can produce the same optical response. This type of mapping cannot be captured by a discriminative network, which can only capture one-to-one or many-to-one relationships. The trained forward ANN reduces the one-to-many mapping issue because it is not an exact surrogate simulator and only approximates the complex, degenerate design space. Tandem networks have been used to design thin film stacks^440^, color filters^441^, and topological photonic structures^442^. To tackle the one-to-many problem, several solutions have also been developed, including conditional generative adversarial networks and conditional variational autoencoders, where a latent vector is introduced in the ANN to address potential multiple solutions^433^.

Discriminative ANNs can also perform inverse design using the backpropagation method. Previously, backpropagation was discussed in of network training, where gradients to neuron weights were calculated to minimize the loss function. To perform inverse design with a trained discriminative ANN using backpropagation, the loss function is defined as the difference between the output and desired optical responses and backpropagation is used to calculate gradients to the input device pattern *x* to minimize the loss function. This method has been used to inversely design scatterers^443^ and photonic crystals^444^. The concept can also be applied to more complex computational graphs that combine ANNs with analytic physical expressions^445^. Furthermore, a neural-adjoint method that adds the boundary loss to the loss function is proposed to improve performance^446,447^.

#### Inverse design with generative ANNs

Deep generative networks^448–450^ are architecturally similar to discriminative networks except that one of the inputs to the network is a latent random variable *z*. As such, the output to the network is a distribution, typically of device patterns, which can be generated by sampling *z*. There are two general inverse-design concepts based on generative networks. The first is to train a generative network to produce distributions that mimic a training set of device patterns. For networks where the input is the desired optical response and *z*, the generator functions as an inverse network: *z* is sampled to produce an ensemble of candidate devices, which are then evaluated and filtered to identify devices with suitable performance. For networks where the input is a device operating parameter (i.e., operating wavelength or material index) and *z*, the generative network can be used for interpolation and output devices with operating parameters not found in the training set.

There are different ways for generative networks to learn training set distributions. One way is with variational autoencoders (VAEs), which consist of two parts: an encoder that maps device patterns onto a low dimensional latent space distribution, and a decoder that maps latent space data representations back to device patterns. The loss function includes a reconstruction term, which minimizes differences between the inputted and outputted device patterns to the full network, and regularization terms that tailor the latent space distribution to fit a Gaussian profile. As shown Fig. 12d, VAEs have been used to design analog power splitters with various splitting ratios^61^, and digital multimode interference waveguides were designed by training a VAE in tandem with a pretrained forward network^451^. Another way to train generative networks is with the generative adversarial network (GAN) framework, where the generative network is trained together with a discriminative classification network that attempts to distinguish whether an input device is fake (i.e., from the generative network) or real (i.e., from the training set). Over the course of training, the discriminator gets better at distinguishing real and fake devices while the generator produces more realistic looking devices. Upon the completion of training, the generator fools a trained discriminator by outputting a distribution of devices mimicking the training set. GANs have been used to design freeform metasurfaces that support tailored spectral responses^448^ and high efficiency meta-gratings^449,450^ (Fig. 12e–g).

A second concept for inverse design with generators is based on the dataless training of generative networks to perform a population-based search for the global optimum^452,453^. These networks are termed global topology optimization networks (GLOnets) and the network training procedure works as follows. Initially, the outputted distribution from the generative network spans the full design space such that sampling z produces a batch of random devices. These devices are evaluated by an EM solver to quantify their performance and performance gradients (i.e., pattern modifications that improve performance), and these performance metrics are used to calculate a special loss function. Back-propagation is used to modify the network based on this loss function, such that the device distribution outputted by the network narrows and gets biased towards higher performance regions of the design space. This process is repeated and the device distribution from the network output distribution eventually collapses, ideally around the global optimum. Initial GLOnet demonstrations were used to optimize metasurface patterns, and the final freeform devices had efficiencies that were consistently better than those designed using gradient-based optimization^452^ (Fig. 12h). GLOnets were also demonstrated to be compatible with reparameterization to enable the global search of metasurfaces with hard minimum feature size constraints^407^. Subsequent improvements to the stability and performance of GLOnets have been made in thin film stack optimization, through judicious selection of the network architecture^453^. These concepts showcase the great potential of hybrid algorithms to interface physics and data sciences.

#### Inverse design with reinforcement learning

In reinforcement learning, a neural network learns to specify a sequence of actions within an environment in a manner that maximizes a cumulative reward^454–456^. These concepts have been popularized with their application to games, such as Atari games or Go, and have had significant technological impact in fields ranging from robotics to communications^457^. By training a deep network to play the game many times, initially with random actions (i.e., exploration) and later with actions informed by past experiences, the network will learn to specify an optimal action that maximizes current and future rewards. The consideration of cumulative rewards during the training process allows the algorithm to balance trade-offs between long-term and short-term reward gains^432^.

The framework of reinforcement learning naturally maps onto the iterative optimization process for nanophotonic devices. In this context, the state is the device pattern at a given iteration, the action is a modification to the pattern, and the reward combines the present and future device performance as quantified by the FOM. As the reinforcement learning algorithm learns from many trajectories through a design landscape and is based on maximizing cumulative reward, the device pattern modification suggested by the algorithm in a given optimization iteration is not simply the modification that produces the largest improvement to the FOM, which is the case in gradient-based optimization. Rather, the algorithm will suggest trajectories through a design landscape that can sidestep local optima in an attempt to maximize the performance of the final device. It is noted that the training process for reinforcement learning is computationally expensive, and the simulation of devices over many trajectories through a design landscape can take days on a conventional CPU, even for relatively basic problems. These algorithms are therefore more practically implemented when paired with accurate surrogate simulators. Reinforcement learning has been used to design periodic dielectric metasurfaces that support tailored spectral responses^454^, multi-layer structures^455^, perfect absorbers^456^, and metasurface holograms^457^ (Fig. 12i).

### Comparisons of different design methods

Table 1 summaries several representative metamaterial waveguides (usually with wavelength-scale dimensions) to briefly compare the inverse-designed methods discussed above. As the landscape of a metamaterial waveguide is generally non- convex, currently it is still not mathematically possible to guarantee global optimal solutions in inverse-design optimization problems^15,58^. Despite that certain global search or reinforcement learning algorithms^47,432,452^ attempt to maximize the performance of the final device, but global optimum still not surely undertaken. Some active research area have been developed to explore the computational bounds on the true optimal value of an given optimization problem, which essentially derive from Lagrange duality^458^, local power conservation^459^ or diagonal physics dual^460^ based on the basic properties of the constraints and objective functions. Such bounds not only help to provide guidelines to the maximal device performance possible within physical limits, but also can be used to rule out constraints and objective functions for which no device pattern can achieve such good objective value.

**Table 1:** Comparisons of different inverse-designed methods for meta-waveguides

Device	Optimization method	Performance metric						Ref.
		Dimensions	Minimum feature	Optical performance				
				Excess Loss	Crosstalk	Bandwidth	Exp.	
3 dB power splitter	Level-set method (Analog)	2.0 × 2.0 µm^2^	/	<0.12 dB	/	100 nm	No	^367^
	DBS (Digital)	2.72 × 2.72 µm^2^	90 nm	0.35 dB (average)	/	60 nm	Yes	^370^
	ANN combined DBS (Digital)	2.6 × 2.6 µm^2^	90 nm	<0.4 dB	/	100 nm	No	^464^
	Adjoint method (Digital)	2.6 × 2.6 µm^2^	70 nm	0.44 dB (average)	/	40 nm	Yes	^62^
2 × 2 power splitter	TO & level-set method (Analog)	3.0 × 1.2 µm^2^	70 nm	0.5 dB (average)	/	45 nm	Yes	^492^
	PSO (Digital)	4.8 × 4.8 µm^2^	100 nm	<3.5 dB	/	25 nm	Yes	^430^
Dual-mode multiplexer	TO (Analog)	2.4 × 4.0 µm^2^	/	<1.2 dB	<−12 dB	100 nm	Yes	^377^
	TO & level-set method (Analog)	3.55 × 2.55 µm^2^	70 nm	<1.0 dB	<−15.6 dB	100 nm	Yes	^492^
	DBS (Digital)	2.4 × 3.0 µm^2^	90 nm	<1.0 dB	<−24 dB	60 nm	Yes	^415^
	Adjoint method (Digital)	2.4 × 3 µm^2^	72 nm	1.51 dB (average)	<−18 dB	40 nm	Yes	^62^
Dual-mode converter	Objective first method (Analog)	1.6 × 2.4 µm^2^	/	0.63 dB	−21.5 dB	@ 1550 nm	No	^58^
	TO & level-set method (Analog)	2.4 × 2.4 µm^2^	80 nm	0.13 dB	/	@ 1550 nm	No	^461^
			160 nm	0.46 dB				
			200 nm	0.97 dB				
	ANN combined DBS (Digital)	3.85 × 2.35 µm^2^	100 nm	<0.71 dB	<−23 dB	100 nm	No	^465^
Wavelength multiplexer	TO & level-set method (Analog)	2.8 × 2.8 µm^2^	100 nm	<1.8 dB (1300 nm)	<−11 dB	100 nm	Yes	^368^
				<2.4 dB (1550 nm)		170 nm		
	DBS (Digital)	2.6 × 5 µm^2^	80 nm	<2.1 dB (1550 nm)	<−16.4 dB	23 nm	Yes	^417^
				<2.3 dB (1573 nm)		18 nm		
Polarization beam-splitter	Density TO (Analog)	1.4 × 1.4 µm^2^	/	<0.82 dB (TE)	<−12 dB (TE)	100 nm	Yes	^376^
				<2.1 dB (TM)	<−15 dB (TM)			
	Objective first method (Analog)	0.48 × 6.4 µm^2^	/	<0.46 dB (TE, TM)	<−14.5 dB (TE, TM)	72 nm	No	^386^
	DBS (Digital)	2.4 × 2.4 µm^2^	120 nm	1.49 dB (TE)	−11.8 dB (TE)	@1550 nm	Yes	^68^
				0.97 dB (TM)	−11.1 dB (TM)			
Chip-fiber grating coupler	ANN & brute-force sweeping (Analog)	/	61.25 nm	3.28 dB (TE, 5° FA)	/	@1560 nm	No	^438^
	PSO (Analog)	/	35.44 nm	3.0 dB (TE, 8.5° FA)				
	DBS (Digital)	15 µm	120 nm	1.54 dB (TE, 0° FA)	/	@1550 nm	No	^533^
	TO & level-set method (Analog)	12 µm	100 nm	4.4 dB (@1514 nm, 5° FA)	/	40 nm	Yes	^394^
				5.3 dB (@1514 nm, 5° FA)		100 nm		
				6.3 dB (@1514 nm, 5° FA)		120 nm		
	PSO (Analog)	12 × 20 µm^2^	67.8 nm	5.8 dB (@1578 nm, 25° FA)	/	90 nm	Yes	534
				4.2 dB (@1578 nm, 25° FA)		48 nm		

*ANN* Artificial neural networks, *Algorithms DBS* direct-binary search, *TO* topology optimization, *PSO* particle swarming optimization, *FA* fiber angle, *Exp* Experimental demonstration.

Gradient-based algorithms (e.g., TO or level-set method) have many advantages, such as faster convergence and powerful mathematics-handling capacity particularly with lager parameter space. However, fabrication constraints are usually necessary. Imposing a larger feature size constraint in the optimization may deteriorate the performance^461^. In some extreme cases, the inverse-design algorithm could not find photonic structures on-demand even without imposing fabrication constraint^462^, the theoretically achievable performance or the desirable functionality may be ultimately bound by material constraints.

Non-gradient-based algorithms (such as DBS, GA or PSO algorithms) are usually easy to implement and need few mathematical requirements (e.g. complex gradient calculations are not needed). However, the convergence is often considerably slower, particularly when the objective function or parameter space becomes complex. To mitigate this issue, parallelizing the algorithm and using larger clusters of processors would be necessary^463^. On the other hand, although digital devices inspired by these non-gradient-based optimization methods has developed rapidly and achieved remarkable successes in many fields, most of the optimization methods are less involve with more complex physics problems, such as some emerging applications in nonlinear optics^366,388,393^.

For deep learning methods, it is possible to accelerate the simulation and design of optical devices in an effective and efficient manner^438,464,465^. The choice of neural network architecture and training strategy depends largely on the type of photonic system being analyzed. For systems that are relatively low dimensional and can be described by fewer than approximately ten independent geometric parameters, a discriminative neural network can effectively serve as a surrogate solver for that system and can be used in various optimization strategies to perform inverse design. These networks do require a large, one-time computational investment into a training set, making the neural network approach appropriate only when it involves systems, such as color filters or meta-atoms, where a wide range of layouts and optical responses are of interest. If a library of useful device shapes is known and are useful guidelines for design, the GAN approach provides a direct route to learning and interpolating related shapes from this library. If the goal is to identify a particularly high-performance device from a computation space, GLOnets provides a strategy to effectively search for the global optimum, without the requirement of a training set.

## Summary and outlook

In summary, optical waveguides have proven a canonical platform to integrate diverse functional subwavelength meta-structures to enable meta-waveguides with either novel functionalities or largely boosted device performance. Compared with conventional waveguides devoid of subwavelength structures, meta-waveguides can not only just guide and confine light, but also perform various functionalities underpinned meta-structures. Compared with photonic crystal waveguides, meta-waveguides can act as ‘designer artificial media’ (with distinctive waveguiding mechanism to photonic bandgap) that enable versatile and powerful control over light propagation with subwavelength precision.

By allying functional subwavelength structures with dielectric and plasmonic waveguide platforms, versatile coupling interfaces, on-chip optical signal processors, photonic neural networks, multifunctional routers, mode convertors, sensors, quantum and nonlinear devices can be envisaged. By integrating meta-structures with optical fibers, massive applications in optical communications, imaging, biomedical sensing and lab-on-fiber technologies can be realized in a flexible, compact and multifunctional manner.

**Fig. 13:**
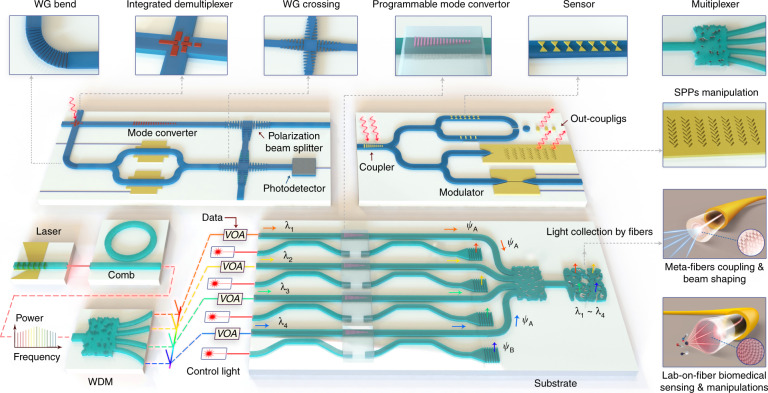
Outlook on photonic integrated “meta-circuits”. Diverse meta-waveguide-based photonic devices can be competitive substitutes for conventional integrated optical scenarios with compact footprint, enhanced efficiency and multifunctionality. A laser-pumped microring frequency comb is distributed to cascaded wavelength division multiplexers (WDM) enabled by inverse-designed metamaterial waveguides (WG). Data can be encoded by variable optical attenuators (VOA) and fed to programmable mode convertors, which can be assembled into integrated photonic networks to perform analog computing of matrix-vector multiplication (MVM) for parallel convolutional processings^177,523^. The reconfigurable mode convertor can convert mode Ψ_A_ to Ψ_B_ (Ψ is TE or TM) with various distinguishable levels or to different spatial mode channels in a multimode waveguide^541^. The optical data are then collected via meta-couplers and meta-fibers with high efficiency and fidelity for further processing. Meta-waveguides can also facilitate on-chip optical signal processing, communications, display and sensing applications (shown as insets). Plasmonic meta-waveguides may shrink device footprint towards higher integration density. Meta-fibers can also find massive interconnects, signal shaping, biomedical sensing and micro-manipulation applications^232,235^. Device schematics are inspired from previous literatures^12,177,194,235,523,541–543^.

**Fig. 14:**
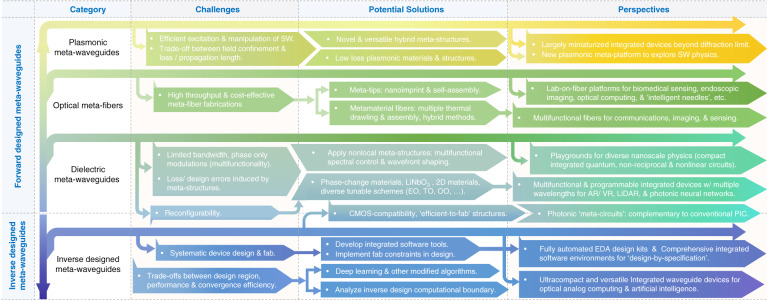
Roadmap. Current challenges, corresponding potential solutions and perspectives are briefly outlined. In the near-term, challenges in systematic device design, multifunctionality and reconfigurability require attention. For further practical applications, compatible integration and efficient fabrication should be properly addressed.

Despite the concept of meta-waveguides is still in its infancy, exciting progress are hatching with bright perspectives and profound potential applications. As conceptually illustrated in Fig. 13, the advancement of meta-waveguides can not only extend meta-optics to the realm of guided electromagnetic waves and waveguide technology, but also promise to reshape the landscapes of photonic integrated circuits and massive emergent applications. The awaiting challenges are outlined as the following. A brief roadmap is also presented as Fig. 14 for future research.

### Challenges

Despite the exciting progress discussed above^12,16,17,466^, practical applications of meta-waveguides are still limited. Several challenges must be properly handled before such ideas may transfer from scientific demonstrations to viable real-word technologies in future.

First, reconfigurable meta-waveguides are highly desired. For most current cases, the device attributes are fixed after fabrication^35–37,41–44,70,75–77^. Further advancement of photonic integrated circuits and other applications will demand multi- functionality and reconfigurability of diverse photonic devices in a way similar to their electronic counterparts^1^. To this end, alliance from material platforms would be necessary. For instance, the meta-structures on top of waveguides can be mold by phase-change materials^177^ or two-dimensional materials^40,467,468^ to facilitate reconfigurable integrated devices. Lithium niobate is also recently emerging as an excellent electro-optic (EO) and nonlinear material platform^469,470^ with new opportunities for creating meta-waveguides. Other tunable mechanisms such as electrical-^32,471^, thermal-^174,472^, mechanical-, and all-optical control may be explored as well^473^.

Second, challenges in device design still exist. (i) For meta-waveguides designed by physical intuitions, a universal design framework is still elusive. Most explorations start from distinct physical models under different waveguide platforms with unique features^51,70,75^. (ii) For inverse-designed meta-waveguides, in scientific research-wise, some academic questions still remain open^15^, such as the existence of solution under given optimization conditions and the ultimate computational boundary. In application-wise, fully automated photonic inverse-design kits and integrated software tools still await development.

Third, as shared by all emergent technologies struggling to migrate from lab to real-word, meta-waveguides also have to settle challenges in scalable fabrication and compatible integration. The meta-structures generally pose stringent requirements on fabrication resolution^12^. Fabrication constraints should be considered in design process to pursue “efficient-to-fabricate structures”. Complementary metal-oxide-semiconductor (CMOS) compatibility to electronics needs to be addressed as well to eventually enable large-scale manufactures fitting the foundry’s process design kits^1^.

Detailed challenges and potential research directions are discussed below, according to different design directions and distinctive waveguide platforms.

#### Challenges for dielectric meta-waveguides

For instance, the substrate light leakage during guided wave and free-space light couplings via metasurface-decorated waveguides will reduce conversion efficiency. This issue could be solved by using waveguide-integrated distributed Bragg reflector substrate, layered structures, and topologically protected structures^474,475^. Besides, the intrinsic dispersive characteristics of the meta-waveguides should be also addressed for broader band operations.

In addition, most of the meta-waveguides introduced above are based on local metasurfaces^146^, where the desired device functionality is realized by independent scatterers ignoring the neighboring coupling of the meta-units^145^. These devices generally operate at single wavelength with phase-only manipulation, for instance, dielectric waveguides patterned with phase- gradient metasurface^35,41–43,51^. To thoroughly handle the detrimental effect of neighboring coupling on device performance and overcome the physical limitations of the local meta-structures^146^, we may need to entail nonlocal metasurfaces^145^.

Nonlocal metasurfaces rely on interactions between adjacent meta-units, showing unique advantages for designing multi- wavelength and multifunctional devices. A novel group theory^145,146^ is proposed to design metasurfaces supporting quasi-bound states in the continuum (q-BICs)^476^, showcasing excellent capability in multifunctional spectral/polarization control, wavefront shaping^145^ and leaky wave phase-amplitude holograms using waveguide-integrated metasurfaces^28,80^.

#### Challenges for optical meta-fibers

One of the salient challenges for meta-structured optical fibers is scalable and cost-effective fiber manufacture with desired subwavelength structures^231,232^. Photonic crystal fibers with comparatively bigger feature size are already commercially on- shelf, yet relevant technologies to massively produce optical fiber meta-tips and metamaterial fibers are still under development. A rich library of templates can be transferred from free-space metasurface to fiber facets. However, currently demonstrated optical fiber meta-tips fabricated by slow and expensive processes such as FIB milling and EBL^75,313,314^ are not suitable for commercial products. Photolithography and femtosecond laser writing on fiber facets and sidewalls require further amelioration on fabrication resolution^290,292,293,305^ and apparatus developments for better sample mounting and alignments^101,313^.

For optical fiber meta-tips, nanoimprint and self-assembly are two promising inroads towards high-throughput fabrication. Despite a sub-15 nm feature size is realized in imprinted fiber facet^291^, the structural quality and robustness still need further improvement^311^. Self-assembly is another cost-effective but ‘bottom-up’ method^477,478^. However, this approach also faces challenges in integrating diverse functional materials on unconventional fiber substrate and the accurate control over assembled structures and locations^233^. Meanwhile, most meta-tips deposit lossy metal on fiber facets^75,100,231^. Other materials that guarantee high light-matter interactions with low loss and fabrication robustness still awaits explorations.

For metamaterial fibers, the current resolution of side-wall photolithography is still not adequate for subwavelength features^316^. Thermal drawling of specific preforms instead offers unique advantages for market exploration. However, challenges such as low spatial resolution and insufficient axial and radial uniformity still recline ahead^246,247^. Although the resolution of a single drawing may be compromised, iterative and multiple drawling can largely reduce feature dimension from microns to tens of nanometers^320,321^. The drawn fibers can be assembled subsequently to produce metamaterial fibers with specific transverse structure distributions^321^. When transferring these techniques to produce optical meta-fibers, structural quality and reproducibility will require further attentions.

#### Challenges for plasmonic meta-waveguides

One long-standing challenge for plasmonic waveguide is lacking an efficient and integrated bridge to couple free-space propagation light into highly confined plasmonic modes^70,332^. On-chip in-coupling gratings can be adopted to convert input light beam to surface plasmons along metal, which is further focused by a taper-like region and then coupled into plasmonic waveguides^479^. Although a large portion of input light has the opportunity to be guided into the waveguide, such solution also suffers from low-efficiency and large-footprint issues. The rapid development of meta-structures offers us a high-efficiency, mini-sized, high-integration and multifunctional platform to manipulate both PWs and SWs^70,112–115^. However, an ideal meta-coupler to efficiently connect free-space optical mode and localized plasmonic waveguide mode is still under research^480^.

Plasmonic meta-waveguides can offer unique capabilities to transport and manipulate light in deep-subwavelength scale, which is especially desired for future highly integrated photonics^324,344^. Nevertheless, the large energy loss (including absorption loss and scattering loss) of highly confined plasmonic waveguide modes is another big issue that seriously hinders their practical applications^481^. To address this challenge, people have reported diverse hybridized meta-waveguides to concentrate more optical energies of plasmonic waveguide modes in low-loss materials (e.g., dielectric layers) instead of conventional lossy metals^340,482–484^. Besides, developing new plasmonic materials (e.g., metallic alloys, doped oxides and semiconductors) also attains intense attentions of the community^481,485^. The propagation length of such hybridized or new-material-based waveguide modes can be further improved^486^. In parallel, the low-cost and high-throughput fabrication techniques for creating high-quality plasmonic devices are always highly desired, which is the very foundation to suppress both kinds of systematic losses.

#### Challenges for Inverse-designed metamaterial waveguides

First, most optimization algorithms become less efficient when processing inverse problem with larger parameter space or big design area^15^. As device complexity increases, new electromagnetic simulation tools and optimization algorithms need to be explored, thereby improving the breadth of inverse-design problems. For Deep learning methods, many reported cases to date are still limited to simple nanophotonic optimization, such as free-space metasurface^439,449,452,457^. Challenges still exist in applying diverse artificial neural networks for designing waveguide-based devices^487^. Metamaterial waveguides generally have more complicated structures and the training dataset acquisition can be more time-consuming. Current explorations are mainly restrained in digital meta-waveguides with simple device functions^61,464,487^. For complex nanostructures with more degrees of freedom (e.g. analog meta-waveguides) or devices with multiple sophisticated functionalities, some pre-processing stages or modification of the model input or output may be required^454,488,489^.

Second, there still lacks a comprehensive physical model to tutor how to choose the design region and initial pattern^153^, especially in rigorous math to answer whether the functionality can be effectively realized or what is the ultimate performance achievable for an optical optimization problem^459^. As the landscape of a meta-structure is generally highly non-convex, a good initial condition is required and the computationally tractable method towards the global optimum of the objective function usually does not exist^153^. Different initial patterns may lead to different local-optimum even using the same iteration algorithm. One has to use multiple random initial patterns to generate many “optimized” patterns and then select a relatively “best” one as the final pattern. For this purpose, several recent works have begun exploring physics-informed optimization methods based on prior physical model, helping to discuss the feasibility of inverse-design quantitatively^415,418,420,421^.

Third, a comprehensive and robust software platform^490,491^ to develop diverse inversely designed integrated devices still needs further development. Standardization in design process is required to accommodate fabrication constraints in commercial photolithography for different types of robust nanophotonic devices^492^.

### Perspectives

Meta-waveguides with subwavelength functional structures offer an exceptional playground to develop novel integrated photonic devices and explore nanoscale optical phenomena^493^. As precise and powerful control over light propagation can be enabled by the meta-structures, light-matter interaction can be further magnified to enhance efficiency. Various emerging device functions still awaits exploring^12^.

#### Playgrounds for nanoscale optical physics and versatile integrated devices

Although in an early phase of research, metas-waveguides have already shown great potentials on manipulating light on-chip with excellent versality^35,50–52^. Their functionalities could be further extended for broader applications. For example, migrating different dynamic light control techniques achieved in free-space metasurfaces^471,473,494^ to meta-waveguides, for various new chip-integrated applications such as augmented/virtual reality (AR/VR) displays and optical ranging/LiDAR^31,32,472^. Beside passive devices, meta-waveguides can be also synergized with active components like waveguide-based lasers (to configure light emitting) and photodetectors^495^ (to enhance the light-matter interaction efficiency).

In addition, there could be further explorations on optical computing with metasurface incorporated waveguides^177^. Integrated photonics have proven ideal optical computing platforms for various tasks, including image processing and artificial intelligence, with unprecedented speed and lower power consumption^191,496^. It is possible that those capabilities can be implemented using either fixed or reconfigurable meta-waveguides for achieving complete on-chip operations with miniaturize device footprint and enhanced multifunctionality^177,497^. Meanwhile, integrated photonic platform may be also further developed to significantly miniaturize conventional free-space optical elements^498,499^. Advanced designs in integrated meta-gratings may also fuel further advancements of diverse applications both in imaging and information processing^192,497^. Moreover, recent advent of quantum silicon photonic chips also open the door towards practical quantum communication and computations with high scalability and compactness^497,500,501^. It is foreseeable that incorporation of meta-photonic structures with photonic integrated quantum chips can achieve more complex functions and bring them to the next level of miniaturization.

Furthermore, if we further engineer the imaginary part of the antennas refractive index (gain & loss) to exploit Parity-Time symmetry^502^ or applying dynamic index modulations^503,504^, the functionalities of the meta-devices can be further extended in a more interdisciplinary manner^502,505–508^.

#### Multifunctional fibers that can sense, image, and communicate

As an important member of meta-waveguide family, optical meta-fibers have shown massive applications in telecommunication, biomedical sensing and so on, by leveraging the flexibility and versatility of meta-fiber-based devices^74,232^.

With the aid of meta-fibers^75,76^ and multi-material fibers^267^, we can envisage multifunctional fibers that can sense^104,232,234,249,294^, image^74,246,276,277^, and communicate^96–98,293^ leveraging versatile subwavelength structures. The optical fibers cease to be just a simple waveguide and become a new all-around technological platform where different kind of materials and structures at nanoscale are suitably integrated around, on top or inside the fiber itself, allowing for the realization of advanced platforms from life science applications and lab-on-fiber (LOF) technology^231^ to optical computing^234^. The revolutionary idea here is to combine all the functional components of a generic optical system commonly employed in communication and sensing fields (light source, optical waveguide, photodetector, module to interact with the environment) into a single, flexible, multifunctional, and compact platform. This perspective will set a fundamental building block for the development of a new generation of portable, ‘plug-and-play’, autonomous optical fiber chips to sense, elaborate and transmit sensorial data in remote locations without needing connection to any bulky instrumentation. The realization of all-in-fiber active optoelectronics platforms will break new grounds in many strategic sectors such as photonic computing and imaging, wearable technology, ‘internet-to-things’ systems and telemedicine^509,510^, by avoiding components such as fiber tapers, grating couplers and bulk lenses that may strongly affect the performances, reliability, and footprint of current lab-on-a-chip devices.

Reliable metasurfaces-assisted ultra-low-loss light delivery and collection may also push optical imaging to a higher level with profound medical and clinical impact. Novel ‘intelligent needles’ can be thus envisaged, for bio-manipulation, tissue and liquid biopsies, loco-regional echography and drug delivery with potential impact in precision medicine scenarios and clinically relevant investigations^511^.

#### On-chip plasmonic meta-devices in deep-subwavelength scale

The capability of confining long-wavelength optical radiations into collective charge oscillations at deep-subwavelength scale has made plasmons as excellent information carriers beyond diffraction limit^150,330^. Compared with conventional dielectric waveguides, plasmonic meta-waveguides can enable ultracompact on-chip optical functional devices of subwavelength dimensions^16,17^. Moreover, a new class of plasmonic meta-waveguide can enable intriguing plasmonic modes beyond those of conventional plasmonic waveguides, such as chiral-^512^, non-diffraction-^123^, complex-polarized-^334^, and conformal- SPPs^344^, providing us a versatile platform to manipulate near-field SWs.

Thanks to the unique characteristics of high speed in time and subwavelength scale in space, plasmonic meta-waveguide- based devices and circuits may serve as competitive complements and ideal links between conventional electric and photonic devices. So far, fruitful results on functional plasmonic meta-devices are keeping emerging, such as laser sources^513^, logic gates^346^, and photodetectors^514^.

Meanwhile, the development of plasmonic waveguides based on diverse new materials also reveals a promising future direction for information communication, optical computation, near-field sensing, electro-optic modulators, and so on. For instance, plasmon polaritons in graphene/hexagonal boron-nitride (h-BN) heterostructures exhibited a long intrinsic propagation length exceeding 10 µm (about 50 plasmonic wavelengths)^515^. Graphene can also be patterned with subwavelength structures to facilitate various meta-waveguide devices for beam steering^516,517^, non-reciprocal SPP propagation^518^ and topological edge plasmon^519^. In addition, plasmonic meta-waveguides carrying strongly enhanced local fields offer us a good platform for tunable light-matter interactions (e.g., nonlinear and Raman effects) when integrated with two-dimensional materials such as MoS_2_ and graphene^348,520^. Other material platforms like doped oxides and semiconductors can be good substitutes to conventional noble metals for high-performance tunable plasmonic devices^481,521^.

#### Smart metamaterial waveguides for photonic computing and deep learning

The long-standing grand challenge in efficient and systematic design of diverse nanophotonic devices may be circumvent by inverse-design methods^15^, which sheds light on integrated optics design automation to enable large-scale sophisticated circuits. Inverse design is especially suitable for developing meta-waveguides-enabled devices, as forward device topology design is much complicated and require specific manpower expertise. As integrated software tools for inverse design are ramping up recently (such as SPINS^490^ and Lumerical Inc.^491^), we can envisage fully automated integrated photonic meta-devices design kits and commercial EDA software in the future, where no special expertise in electrodynamics or integrated photonics is required in this ‘design-by-specification’ scheme. Standardization and system level integration will be entailed in later stage as helpful catalyzer to push integrated meta-waveguide devices one step closer to real-word applications.

As fruitful results are keep emerging recently with enhanced device performance and even previously inaccessible novel functionalities^387,422^, inverse-designed metamaterial waveguides may hatch as a significant complement to integrated photonics mainstreams designed by physical intuitions^395^. A new twist in optical analog computing may take place, by shrinking bulky systems into elements in mere wavelength-size^57,522^. The metamaterial waveguide itself can be designed as ‘smart’ media for photonic computing and artificial intelligence^497,523^, for realizing parallel mathematical operations (such as differentiation, integration, or convolution)^57,524^, object classification^59,525^, image analysis^526^, or feature detection^527^. In the meantime, inverse-designed meta-waveguides can also find its place in deep learning and neuromorphic photonics^8,148^ to facilitate chip-integrated high-speed photonic neural networks with low power consumption^60,528,529^. For instance, a metamaterial can be trained to perform vowel recognition for acoustic wave^60^. Analog computing can be thus implemented in a manner that relies on purposefully perturbing a given system in situ, instead of designing the material from scratch, showcasing applications in all-optical control^428^ as well as programmable meta-inclusions in the microwave regime^530^. Programmable meta-units are also explored as physical weights in an end-to-end deep learning integrated sensing pipeline to enable joint learning of optimal measurement process and a matching processing algorithm with improved latency^531^. With the aid of tunable materials, for instance phase-change materials and EO materials, meta-waveguides can venture photonic integrated circuits into new territories by providing diverse integrated devices with multifunctionality and reconfigurability^177,532^.
